# Novel synthetic pathway for the production of phosgene

**DOI:** 10.1126/sciadv.abj5186

**Published:** 2021-09-29

**Authors:** Patrick Voßnacker, Alisa Wüst, Thomas Keilhack, Carsten Müller, Simon Steinhauer, Helmut Beckers, Sivathmeehan Yogendra, Yuliya Schiesser, Rainer Weber, Marc Reimann, Robert Müller, Martin Kaupp, Sebastian Riedel

**Affiliations:** 1 Freie Universität Berlin, Institut für Chemie und Biochemie–Anorganische Chemie, Fabeckstr. 34-36, D-14195 Berlin, Germany.; 2 Covestro Deutschland AG, 51365 Leverkusen, Germany.; 3 Technische Universität Berlin, Institut für Chemie Theoretische–Chemie, Straße des 17, Juni 135, D-10623 Berlin, Germany.

## Abstract

Chloride ions are efficient catalysts for the synthesis of phosgene from carbon monoxide and elemental chlorine at room temperature and atmospheric pressure. Control experiments rule out a radical mechanism and highlight the role of triethylmethylammonium trichloride, [NEt_3_Me][Cl_3_], as active species. In the catalytic reaction, commercially available [NEt_3_Me]Cl reacts with Cl_2_ to form [NEt_3_Me][Cl_3_], enabling the insertion of CO into an activated Cl─Cl bond with a calculated energy barrier of 56.9 to 77.6 kJ mol^−1^. As [NEt_3_Me]Cl is also a useful chlorine storage medium, it could serve as a catalyst for phosgene production and as chlorine storage in a combined industrial process.

## Teaser

A new approach for the production of the industrially important intermediate phosgene based on chloride catalysis was developed.

## INTRODUCTION

Since its discovery in 1812 by Davy (*1*), phosgene [C(O)Cl_2_] has evolved as one of the most important industrial chemicals along with, sulfuric acid, ammonia, ethylene, and chlorine. As an “intermediate” chemical, it serves as starting material for polymers, agrochemicals, and pharmaceuticals to mention only a few (*2*). Currently, 12 million metric tons are produced per year mainly for the synthesis of polyurethanes and polycarbonates, and it is estimated that the production of phosgene will rise to 18.6 million metric tons/year until 2030 (*2*). Because of its high toxicity, phosgene is only manufactured by a few specialized companies, typically on multi-ton scale requiring a multilevel safety concept. It is obtained by gas phase reaction of carbon monoxide and chlorine at elevated temperature using activated carbon as a catalyst (Eq. 1) (*2*)

$${\text{CO}}_{\left(g\right)}+{\text{Cl}}_{2(g)}\overset{\text{cat}.}{⇌}C(O){\text{Cl}}_{2(g)}$$ (1)

Although the exact mechanism of the phosgene formation is still under debate, it is widely accepted that the reaction is initiated by activation of the Cl─Cl bond. As proposed by Lennon and co-workers (*3*), the first step is a dissociative chemisorption of Cl_2_ on the carbon surface. The adsorbed chlorine atoms react with gaseous CO (Eley-Rideal mechanism) to form a surface-bound acyl chloride entity [C(O)Cl_(ad)_], which further reacts with a surface-bound chlorine atom, leading to phosgene (Langmuir-Hinshelwood mechanism). In contrast, Lercher and co-workers (*4*) assume a two-step Eley-Rideal mechanism, when C_60_ fullerene is used as model system, while the catalytically active species is the surface-bound triplet diradical [C_60_···Cl_2_]^••^. Further studies on nitrogen-modified carbon materials propose a polarization of the Cl─Cl bond by interaction with electropositive carbon sites of the material (Lewis acid catalysis). Reaction with CO leads to an acyl chloride cation [C(O)Cl^+^] and a weakly bound Cl^−δ^, which react with each other to form phosgene (*5*).

The carbon-based catalysts lower the activation energy for phosgene formation to relatively low values of 32 to 56 kJ mol^−1^ (*4*, *6*–*8*). However, the high exothermicity of the reaction (Δ*H* = −107.6 kJ mol^−1^) and subsequent dissipation of process heat is more problematic as the temperature in the iron tube reactors can rise up to 550°C at hotspot reaction sites (*2*). Because of the high temperatures, the catalysts also slowly degrade by attack of Cl_2_ and Cl^•^ atoms on carbon defects, leading to the corrosive formation of HCl and CCl_4_, which leads to shorter maintenance cycles of the reactor (*7*, *9*).

At the outset of this work, we anticipated that the reaction of CO and Cl_2_ to C(O)Cl_2_ could be catalyzed by activation of Cl_2_ using a weakly coordinated chloride anion. In the reaction of Cl^−^ and Cl_2_, polychlorides are formed, which show a broad structural diversity and promising applications (*10*).

The bonding properties of various trichlorides, the simplest polychlorides, were recently analyzed by experimental and computed electron density studies. Accordingly, there seems to be a smooth transition from an asymmetric [Cl…Cl─Cl]^−^ unit to a symmetric [Cl─Cl─Cl]^−^ anion with two equal Cl─Cl bonds in a crystalline environment. These different bonding types of the [Cl_3_]^−^ anion are crucial for its chemical reactivity (*11*).

Depending on the cation, the trichloride [Cl_3_]^−^ is a yellowish salt or a room temperature ionic liquid (RT-IL). Alkylammonium salts such as triethylmethylammonium chloride, [NEt_3_Me]Cl, are considered to be potential materials for the efficient and convenient storage of elemental chlorine as an RT-IL at atmospheric pressure (*12*). This could enable a more flexible chlorine production that can be adapted to the availability of (renewable) electrical energy and thus represents a secondary energy storage system. Here, we report a preparation of phosgene from carbon monoxide and elemental chlorine that proceeds in a homogeneous reaction at room temperature and atmospheric pressure using a [NEt_3_Me]Cl/[NEt_3_Me][Cl_3_] catalyst system.

## RESULTS AND DISCUSSION

**Fig. 1.**
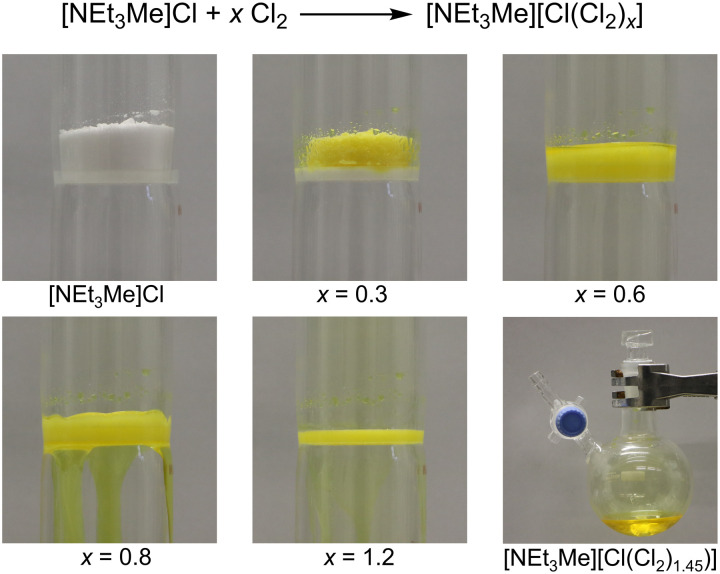
Treatment of solid [NEt_3_Me]Cl with elemental chlorine afforded a stable yellowish RT-IL. Photo credit: Patrick Voßnacker, FU Berlin.

First, [NEt_3_Me]Cl was reacted with elemental chlorine gas, forming [NEt_3_Me][Cl_3_] and higher polychlorides. The amount of absorbed chlorine can be expressed by the general sum formula [NEt_3_Me][Cl(Cl_2_)*_x_*] where *x* depends on the partial pressure of chlorine and the size of *x* has a great influence on the properties of the system (see the Supplementary Materials). When *x* < 0.8, the system exists as a yellow solid, while an increase of the chlorine concentration (*x* > 0.8) results in the formation of an RT-IL (see Fig. 1 and movie S1).

Initially, the stoichiometric reaction of CO with liquid [NEt_3_Me][Cl(Cl_2_)*_x_*] (*x* = 1.1) to C(O)Cl_2_ at room temperature was investigated by gas-phase infrared (IR) spectroscopy, indicating rapid formation of phosgene. If by consumption of Cl_2_ *x* becomes smaller than 0.8, [NEt_3_Me][Cl(Cl_2_)*_x_*] starts to solidify, which results in a decreased reaction rate. Thus, [NEt_3_Me][Cl(Cl_2_)*_x_*] was dispersed in *ortho*-dichlorobenzene (*o*DCB), a standard solvent in phosgene manufacturing. Using an excess of CO (2.3 equiv.), a quantitative conversion of [NEt_3_Me][Cl(Cl_2_)*_x_*] to C(O)Cl_2_ and [NEt_3_Me]Cl was observed. An excess of CO avoids chlorine contamination of the phosgene product, which is difficult to remove, whereas separation of CO and C(O)Cl_2_ is industrial praxis. CO consumption proceeds with a half-life time of 287 ± 14 min (see the Supplementary Materials).

Because the reaction of [NEt_3_Me]Cl and Cl_2_ is very fast, we envisaged a catalytic process by in situ regeneration of [NEt_3_Me][Cl(Cl_2_)*_x_*]. Even with a relatively low catalyst loading of 3.5 mole percent (mol %) [NEt_3_Me]Cl, full conversion of Cl_2_ to phosgene was achieved.

**Fig. 2.**
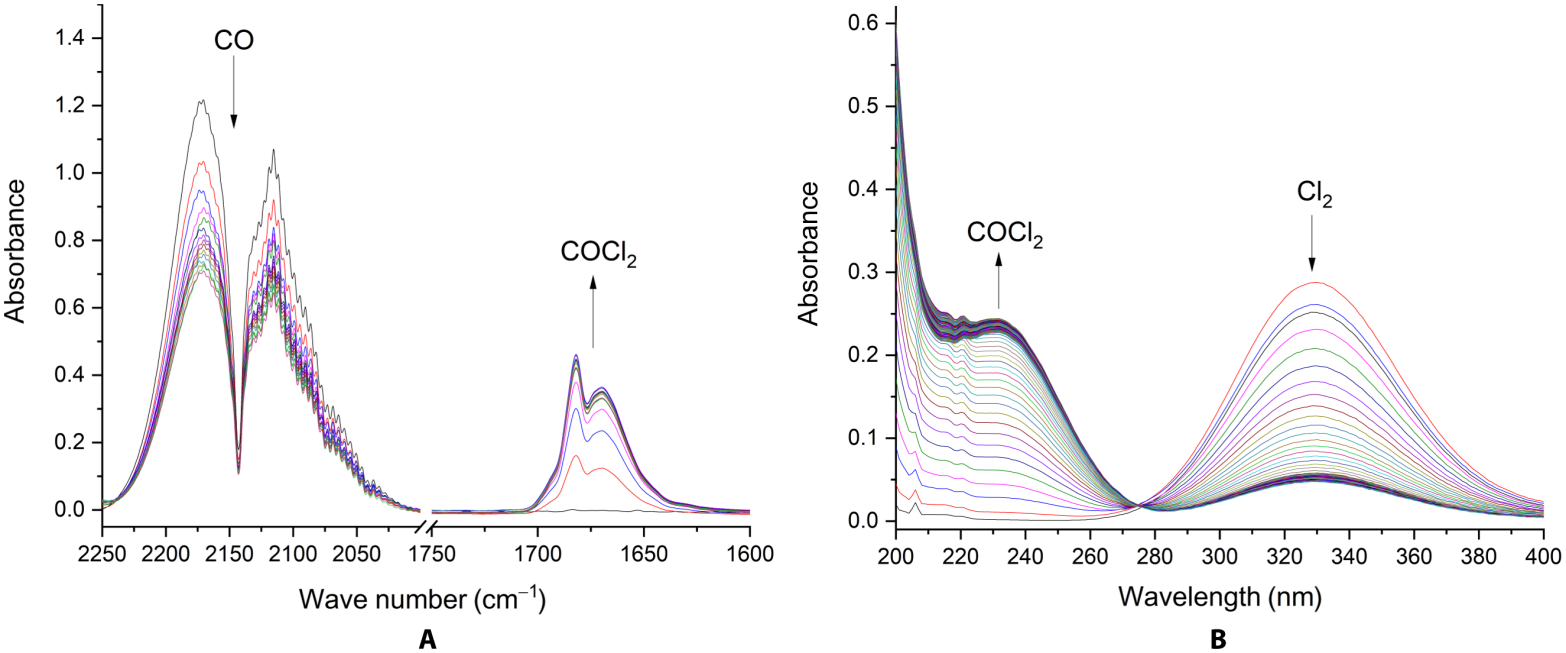
Monitoring the reaction of CO with [NEt_3_Me][Cl(Cl_2_)*_x_*] (*x* = 1.45). IR (**A**) and UV spectra (**B**) were recorded for 420 min and are shown with 30-min (IR) and 5-min intervals (UV).

To improve the contact time between liquid and gaseous reactants, we designed a flow setup, which consists of a gas-washing bottle, filled with a dispersion of [NEt_3_Me][Cl(Cl_2_)*_x_*] in *o*DCB, and a peristaltic pump for continuously circulating gaseous CO and already formed C(O)Cl_2_ in the system (see figs. S1 and S2). This setup allows a spectroscopic in situ monitoring of the reaction progress by passing the gas mixture through IR and ultraviolet/visible (UV/Vis) cells. The consumption of CO (IR spectrum; Fig. 2A) and Cl_2_ (UV/Vis spectrum; Fig. 2B) as well as a simultaneous formation of C(O)Cl_2_ were recorded, indicating an immediate start of the reaction.

**Fig. 3.**
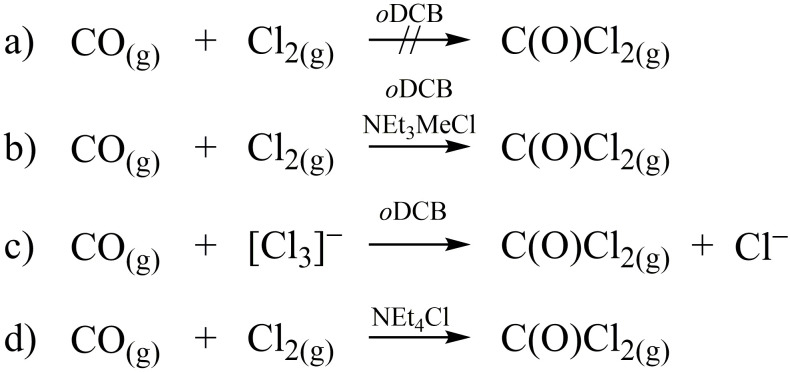
Control reactions of CO with Cl_2_ and [NEt_3_Me][Cl_3_] or catalyzed by chloride salts.

Control experiments with Cl_2_ and CO in our setup have shown that both the beam of the UV/Vis spectrometer and visible light slightly contribute to the formation of C(O)Cl_2_ by photolytic activation of Cl_2_. Therefore, all experiments were conducted in the dark to avoid light-induced radical formation and to emulate industrial processing of phosgene in stainless steel tubes. To demonstrate that the phosgene formation requires no photoactivation, we conducted a series of control experiments. CO was treated with Cl_2_ without a chloride salt (**a**), in the presence of catalytic amounts of solid [NEt_3_Me]Cl (**b**), and with liquid [NEt_3_Me][Cl(Cl_2_)*_x_*] (*x* = 1.45), to mimic the use of a chlorine storage medium, instead of Cl_2_ (**c**; see Fig. 3 and the Supplementary Materials). In experiment **a**, no formation of phosgene was observed in the dark, but in experiments **b** and **c**, C(O)Cl_2_ was formed (see fig. S22), highlighting the involvement of [NEt_3_Me][Cl_3_] as reactive species. In addition, [NEt_4_]Cl, which forms at room temperature the solid trichloride salt [NEt_4_][Cl_3_], was applied as another catalyst to investigate whether a solid/gas reaction could be used for the production of phosgene (**d**). Using this catalyst, formation of C(O)Cl_2_ was substantially slower compared to reaction **b** (see the Supplementary Materials). Notably, no attack of the cation [NEt_3_Me]^+^ in [NEt_3_Me][Cl(Cl_2_)*_x_*] (*x* = 1.6) was observed, when stored under an atmosphere of chlorine gas at 1 bar at room temperature for years, as shown by Raman spectroscopy.

To rule out a radical mechanism, we treated the ionic liquid [NEt_3_Me][Cl_3_] with methane in the dark. As neither chlorinated methane nor HCl was observed IR spectroscopically (for details, see the Supplementary Materials), a radical-based mechanism for the formation of phosgene under these conditions was rejected.

**Fig. 4.**
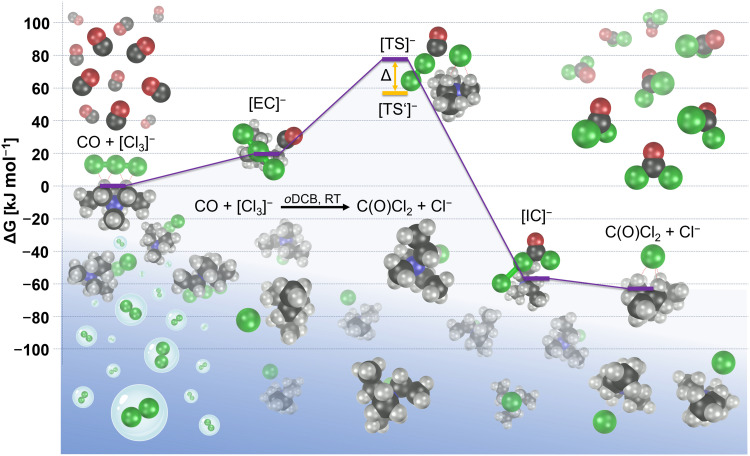
Calculated pathway for the reaction of CO and [Cl_3_]^−^ to C(O)Cl_2_ and Cl^−^. The calculated energies include zero-point energy correction, temperature effects at 298.15 K, solvent effects for *o*DCB, and one [NEt_3_Me]^+^ cation. [EC]^−^, encounter complex; [TS]^−^, transition state including one [NEt_3_Me]^+^ cation; [TS′]^−^, transition state without any cation; [IC]^−^, intermediate complex; RT, room temperature.

To achieve further mechanistic understanding, we carried out quantum-chemical CCSD(T)-F12 single-point energy calculations based on spin-component-scaled (SCS) second-order Møller–Plesset perturbation theory (MP2) structure optimizations using the conductor-like screening model for realistic solvents (COSMO-RS) solvent model for *o*DCB and with or without inclusion of one [NEt_3_Me]^+^ cation (see computational details). Gibbs free reaction energies were computed from the electronic energies at these levels and thermal contributions obtained from normal mode analyses at SCS-MP2 level. On the basis of these calculations, the reaction is initiated by the formation of a very weakly bound encounter complex of CO and [Cl_3_]^−^ ([EC]^−^ in Fig. 4; with closest C─Cl distances of about 3.4 Å). Shortening of C─Cl distances leads to a transition state ([TS]^−^) with a relative Gibbs free energy of 78 kJ mol^−1^ (including one [NEt_3_Me]^+^ cation) or 57 kJ mol^−1^ (neglecting cations), which can be described as a chloride ion loosely bound to an almost linear O─C─Cl─Cl moiety [Cl^−^···C(O)─Cl─Cl]. Charge transfer from the chloride to the O─C─Cl─Cl moiety leads to a [ClC(O)Cl···Cl]^−^ intermediate complex, ([IC]^−^ in Fig. 4), which spontaneously dissociates into C(O)Cl_2_ and Cl^−^. In summary, the reaction of CO and [Cl_3_]^−^ can be regarded as the insertion of CO into an activated Cl─Cl bond, leading to C(O)Cl_2_ and Cl^−^ by releasing about 60 kJ mol^−1^.

However, the computational estimation of the reaction barrier depends on cation-anion interactions. When the influence of only one cation is taken into account, the free energy of the transition state is relatively high (77.6 kJ mol^−1^). In contrast, when no cation effects but only solvent effects are considered, the transition state is calculated to have a substantially lower energy (56.9 kJ mol^−1^). As the real system involves the interaction of multiple cations and solvent molecules, the real free energy of the transition state can be expected to be in the same range between 56.9 and 77.6 kJ mol^−1^. For the uncatalyzed reaction of CO and Cl_2_ to phosgene, a free energy activation barrier of about 230 kJ mol^−1^ was calculated. This indicates that the activation barrier is tremendously reduced by our chloride catalyst comparable to the activated carbon–catalyzed process.

**Fig. 5.**
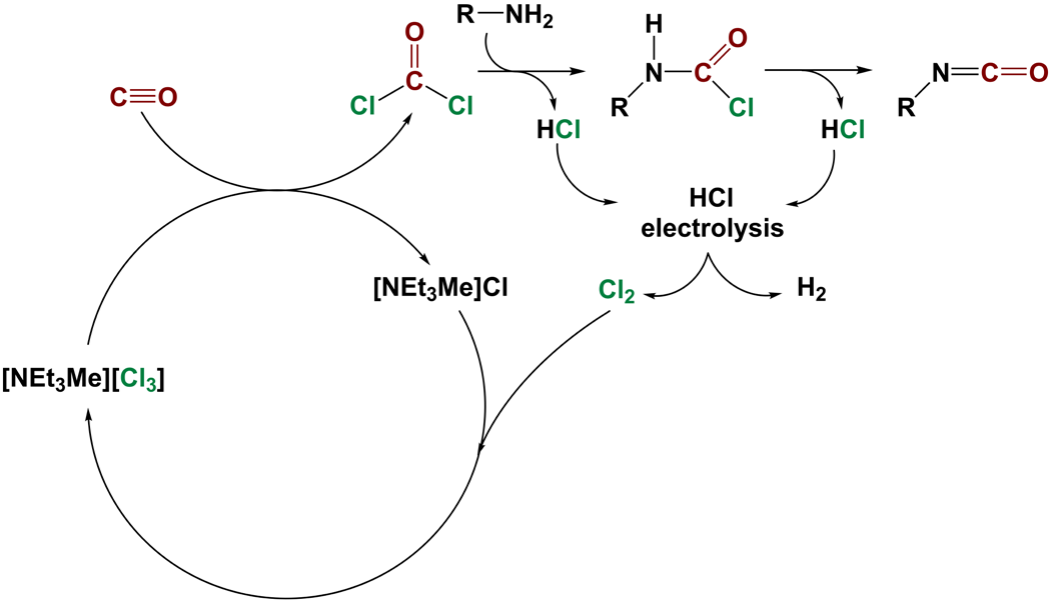
Proposed scheme for a phosgene synthesis using [NEt_3_Me]Cl as a catalyst coupled with a subsequent phosgenation of amines and chlorine regeneration.

On the basis of these results, a catalytic reaction scheme can be proposed in which phosgene is prepared at room temperature by chloride catalysis. The prepared phosgene can be used for further processes, most importantly the synthesis of isocyanates to produce polyurethanes. This was demonstrated for the synthesis of phenyl isocyanate by adding aniline to the generated phosgene solution (see the Supplementary Materials). In the reaction of amines with phosgene to isocyanates, HCl is released, which, in an industrial process, is to some extent typically electrolyzed to regenerate elemental chlorine (Fig. 5).

Concluding, a new synthesis of phosgene was developed, enabled by the main-group catalyst [NEt_3_Me]Cl. As an active intermediate, the ionic liquid [NEt_3_Me][Cl_3_] reacts with carbon monoxide at room temperature and atmospheric pressure to C(O)Cl_2_ in an ionic process. Quantum-chemical calculations suggest an insertion of CO into an activated Cl_2_ moiety with a relatively low barrier between 56.9 and 77.6 kJ mol^−1^. The unique property of [NEt_3_Me]Cl to serve both as a convenient chlorine storage medium and as an efficient catalyst for the production of phosgene opens up new industrial options.

## MATERIALS AND METHODS

### Apparatus and materials

All substances sensitive to water and oxygen were handled under an argon atmosphere using standard Schlenk techniques and oil pump vacuum up to 10^−3^ mbar. Dry *o*DCB was obtained after storage over activated 3-Å molecular sieves. Commercially available triethylmethylammonium chloride (TCI) and tetraethylammonium chloride (TCI) were dried in vacuo at 150°C for 1 hour before use. Aniline (Acros), chlorine (5.0, Linde), and carbon monoxide (2.0, Linde) were used without further purification. Raman spectra were recorded on a Bruker (Karlsruhe, Germany) MultiRAM II equipped with a low-temperature Ge detector (1064 nm, 100 to 180 mW, resolution of 4 cm^−1^). IR spectra were recorded on a Nicolet iS5 Fourier transform IR (FTIR) spectrometer (gas IR cell: 10 cm, KBr windows) or a Bruker Vector 22 FTIR spectrometer (gas IR cell: 10 or 20 cm, silicon windows). UV/Vis spectra were recorded on a PerkinElmer Lambda 465

$$\left[{\text{NEt}}_{3}\text{Me}][{\text{Cl}}_{3}]+\text{CO}\to C(O){\text{Cl}}_{2}+[{\text{NEt}}_{3}\text{Me}]\text{Cl}(\text{Batch}\right)$$

[NEt_3_Me]Cl (0.371 g, 2.446 mmol, 0.35 equiv.) was loaded into a 500-ml Schlenk flask, dried in vacuo at 150°C for 1 hour, and suspended in 1.5 ml of *o*DCB. The solution was degassed, and chlorine was added until the system retained a pressure of 200 mbar (0.493 g, 6.953 mmol, 1 equiv.). CO (800 mbar) (ca. 16 mmol, 2.3 equiv.) was added to the flask, and the reaction mixture was stirred for 2 days in the dark. To isolate the C(O)Cl_2_, the flask was cooled to −15°C, and volatile constituents were distilled in vacuo in two cooled traps held at −60°C (*o*DCB) and −196°C [C(O)Cl_2_, Cl_2_]. Phosgene was transferred into a pressure-stable Schlenk tube, weighted (0.710 g, 7.178 mmol, 103%), and identified by its known Raman (Raman spectrum shows no bands of Cl_2_; fig. S5) and IR spectra (fig. S6).

IR (gas phase): $\tilde{\nu }$ = 2349 (CO_2_), 1827 (s), 1675 (w), 1409 (vw), 1007 (w), 849 (vs), 580 cm^−1^ (w).

Raman (liquid): $\tilde{\nu }$ = 1809 (m), 832 (vw), 571 (vs), 444 (m), 301 cm^−1^ (m)

$$3.5\;\text{mol}\%[{\text{NEt}}_{3}\text{Me}]\text{Cl}+{\text{Cl}}_{2}+\text{CO}\to C(O){\text{Cl}}_{2}+[{\text{NEt}}_{3}\text{Me}]\text{Cl}$$

[NEt_3_Me]Cl (0.053 g, 0.351 mmol, 0.035 equiv.) was loaded into a 500-ml Schlenk flask, dried in vacuo at 150°C for 1 hour, and suspended in 20 ml of *o*DCB. The solution was degassed, and chlorine was added until the system retained a pressure of 200 mbar (0.700 g, 10.009 mmol, 1 equiv.). CO (800 mbar) (ca. 16 mmol, 1.6 equiv.) was added to the flask, and the reaction mixture was stirred for 7 days in the dark. To isolate the C(O)Cl_2_, the flask was cooled to −15°C, and all volatile constituents were distilled in vacuo in two cooled traps held at −60°C (*o*DCB) and −196°C [C(O)Cl_2_, Cl_2_]. Phosgene was transferred into a pressure-stable Schlenk tube, weighted (1.006 g, 10.272 mmol, 103%), and identified by its IR spectrum (fig. S7).

IR (gas phase): $\tilde{\nu }$ = 2349 (CO_2_), 1827 (s), 1675 (w), 1409 (vw), 1007 (w), 849 (vs), 580 cm^−1^ (w)

$$\left[{\text{NEt}}_{3}\text{Me}]\text{Cl}+x{\text{Cl}}_{2}\to [{\text{NEt}}_{3}\text{Me}][\text{Cl}{\left({\text{Cl}}_{2}\right)}_{x}\right]$$

[NEt_3_Me]Cl (10.1 g, 66.5 mmol) was dried in vacuo at 150°C for 1 hour. Chlorine was added until the system retained a pressure of 800 mbar (6.9 g, 97.3 mmol, 1.45 equiv.). A yellow liquid was obtained, which was identified by its Raman spectrum (fig. S8).

Raman (liquid): $\tilde{\nu }$ = 3022 (w), 2988 (s), 2947 (s), 1456 (w), 1071 (vw), 681 (w), 454 (s), 276 cm^−1^ (vs).

### General description of the flow setup

To investigate the kinetics of the reaction between [NEt_3_Me][Cl_3_] and CO, a glass vacuum line was connected to a gas-washing bottle (reactor) via a peristaltic pump, which successively circulates gaseous reactants through the reactor, a UV/Vis, and an IR flow cell, to monitor the formation of C(O)Cl_2_ and the consumption of Cl_2_ and CO as well (see figs. S1 and S2). To ensure proper mixing of all reactants, the liquid and solid reactants were filled into the gas-washing bottle, and all gaseous reactants are pumped through the system. All connections were made using either perfluoroalkoxy alkane or C-Flex Ultra (Cole-Parmer) tubing. This setup was used for experiments using stoichiometric and varying catalytic amounts of [NEt_3_Me]Cl for the reaction of CO + Cl_2_. In addition, blind experiments for the noncatalyzed reaction between Cl_2_ and CO were performed, which showed that the beam of the UV/Vis spectrometer and visible light can induce the formation of phosgene from Cl_2_ and CO. Therefore, all following experiments have been performed in the dark (see fig. S3). Also, the reaction between [NEt_3_Me]Cl, Cl_2_, and CH_4_ was studied to verify a nonradical mechanism. The progress of the reaction was monitored by integrated absorbances of the IR bands in the spectral regions between 1995 and 2250 cm^−1^ (CO) and 1760 and 1885 cm^−1^ [C(O)Cl_2_]

$$\left[{\text{NEt}}_{3}\text{Me}]\text{Cl}+{\text{Cl}}_{2}+\text{CO}\to C(O){\text{Cl}}_{2}+[{\text{NEt}}_{3}\text{Me}]\text{Cl}(\text{flow}\right)$$

[NEt_3_Me]Cl (0.460 g, 3.033 mmol) and 20 ml of *o*DCB were filled into a gas-washing bottle, which was connected to a glass vacuum line and an IR flow cell. The reaction mixture was degassed, and the system was filled with a mixture of CO and Cl_2_ (CO: 1000 mbar, 22.32 mmol, 553 ml; Cl_2_: 208 mbar, 4.65 mmol, 553 ml). The gaseous reactants were pumped through the system using a peristaltic pump for 226 min. IR spectra were recorded after 0, 6, 11, 16, 21, 26, 31, 36, 41, 46, 51, 56, 61, 66, 71, 76, 81, 86, 91, 96, 101, 106, 111, 116, 121, 126, 131, 136, 141, 184, 191, 196, 201, 206, 211, 216, 221, and 226 min (figs. S9 to S11)

$$\left[{\text{NEt}}_{3}\text{Me}][{\text{Cl}}_{3}]+\text{CO}\to C(O){\text{Cl}}_{2}+[{\text{NEt}}_{3}\text{Me}]\text{Cl}(\text{flow}\right)$$

[NEt_3_Me][Cl(Cl_2_)_1.50_] (0.783 g, 3.033 mmol [NEt_3_Me]Cl + 4.555 mmol Cl_2_) and 20 ml of *o*DCB were filled into a gas-washing bottle, which was connected to a glass vacuum line and an IR flow cell. The reaction mixture was degassed, and the system was filled with CO (1000 mbar, 553 ml, 22.32 mmol). The gaseous reactants were pumped through the system using a peristaltic pump for 153 min. IR spectra were recorded after 0, 5, 10, 15, 20, 25, 30, 35, 40, 45, 50, 55, 60, 65, 70, 75, 80, 85, 98, 103, 108, 113, 123, 128, 133, 138, 144, 154, 164, 174, 184, 194, 204, 214, and 224 min (figs. S12 to S14).

[NEt_3_Me][Cl(Cl_2_)_1.47_] [3.904 g, 15.30 mmol (22.49 mmol Cl_2_)] and 20 ml of *o*DCB were filled into a gas-washing bottle, which was connected to a glass vacuum line, an IR flow cell, and a UV/Vis flow cell. The reaction mixture was degassed, and the system was filled with CO (1000 mbar, 800 ml, ca. 32 mmol). The gaseous reactants were pumped through the system using a peristaltic pump for 420 min. IR and UV spectra were recorded every 5 min

$$\left[{\text{NEt}}_{4}]\text{Cl}+{\text{Cl}}_{2}+\text{CO}\to C(O){\text{Cl}}_{2}+[{\text{NEt}}_{4}]\text{Cl}(\text{flow}\right)$$

[NEt_4_]Cl (0.230 g, 1.393 mmol) was filled into a gas-washing bottle, which was connected to a glass vacuum line and an IR flow cell. The system was evacuated and filled with a mixture of CO and Cl_2_ (CO: 850 mbar, 20.20 mmol, 588 ml; Cl_2_: 150 mbar, 3.530 mmol, 588 ml). The gaseous reactants were pumped through the system using a peristaltic pump for 628 min. IR spectra were recorded after 0, 1, 6, 11, 21, 27, 32, 37, 42, 47, 52, 65, 77, 137, 197, 257, 317, 377, 437, 497, 558, and 628 min (figs. S15 to S17)

$${\text{Cl}}_{2}+\text{CO}\to C(O){\text{Cl}}_{2}(\text{flow})$$

*o*DCB (20 ml) was filled into a gas-washing bottle, which was connected to a glass vacuum line and an IR flow cell. The *o*DCB was degassed, and the system was filled with a mixture of CO and Cl_2_ (CO: 850 mbar, 20.20 mmol, 588 ml; Cl_2_: 150 mbar, 3.53 mmol, 588 ml). The gaseous reactants were pumped through the system using a peristaltic pump for 811 min. IR spectra were recorded after 0, 4, 9, 14, 18, 19, 34, 49, 64, 79, 94, 109, 124, 139, 154, 261, 262, 323, 384, 445, 506, 567, 628, 689, 750, and 811 min (figs. S18 to S20). Repetition of the experiment without exclusion of light yields the formation of phosgene, which was shown by gas-phase IR spectroscopy (fig. S21)

$$\left[{\text{NEt}}_{3}\text{Me}]\text{Cl}+{\text{Cl}}_{2}+{\text{CH}}_{4}\to {\text{CH}}_{3}\text{Cl}+[{\text{NEt}}_{3}\text{Me}][{\text{HCl}}_{2}](\text{flow}\right)$$

[NEt_3_Me]Cl (0.499 g, 3.290 mmol) and 20 ml of *o*DCB were filled into a gas-washing bottle, which was connected to a glass vacuum line and an IR flow cell. The reaction mixture was degassed, and the system was filled with a mixture of argon, CH_4_, and Cl_2_ (argon: 705 mbar, 588 ml; CH_4_: 95 mbar, 588 ml, 2.25 mmol; Cl_2_: 200 mbar, 4.74 mmol, 588 ml). The gaseous reactants were pumped through the system using a peristaltic pump for 242 min. IR spectra were recorded 0, 3, 6, 9, 12, 15, 19, 22, 25, 28, 39, 40, 100, 161, 221, 282, 342, 403, 363, and 524 min (fig. S23).

### Proof for the formation of phenyl isocyanate

[NEt_3_Me]Cl (0.367 g, 2.420 mmol, 0.31 equiv.) was loaded into a 500-ml two-neck Schlenk flask, dried in vacuo at 150°C for 1 hour, and suspended in 20 ml of *o*DCB. The solution was degassed, and chlorine was added until the system retained a pressure of 200 mbar (0.560 g, 7.898 mmol, 1 equiv.). CO (800 mbar) (ca. 16 mmol, 2.3 equiv.) was added to the flask, and the reaction mixture was stirred for 3 days in the dark. The reaction mixture was cooled to −196°C and degassed. Then, the system was filled with dry argon gas and connected to a dropping funnel and a condenser that was cooled by using −15°C cold ethanol. The condenser was opened to the fume hood via a gas bubbler and a series of four gas-washing bottles, two of which are filled with a KOH solution and an NH_4_OH solution, respectively, each followed by an empty bottle. A solution of 0.5 ml of aniline (0.51 g, 5.476 mmol, 0.69 equiv.) in 5 ml of *o*DCB was added slowly via the dropping funnel to the reaction mixture held at −15°C, and the reaction mixture was then heated to 100°C for 8 hours. After that, excess of phosgene was removed in vacuo, and the analysis of the reaction products using gas-phase IR spectroscopy revealed the characteristic NCO stretching band at 2273 cm^−1^ of the reaction product (fig. S24).

### Computational details

Structure optimizations at density functional theory (DFT) and SCS-MP2 (*13*) levels, as well as with the ONIOM (*14*) procedure to mix quantum-chemical levels, were performed with the Gaussian 16 program, Revision A.03 (*15*). Structure optimizations at the SCS-MP2 level were enabled by specifying IOp(3/125=0333312000) in the root section of the input file during MP2 optimizations. Additional single-point SCS-MP2 and CCSD(T)-F12 calculations were performed with the Molpro program, version 2019.1 (*16*, *17*). For all atoms of the CO + [Cl_3_]^−^ system, aug-cc-pVTZ basis sets (*18*–*20*) were used. For all atoms in the [NEt_3_Me]^+^ cation, smaller cc-pVTZ basis sets were applied in the second layer of the ONIOM calculations (see below), while full aug-cc-pVTZ basis sets were used for the entire system in subsequent SCS-MP2 single-point energy calculations. For the CCSD(T)-F12 calculations, the associated default auxiliary basis sets (cc-pVTZ-JKFIT, aug-cc-pVTZ-JKFIT, and aug-cc-pVTZ-MP2FIT) (*21*) were used. Solvent effects were included during the structure optimizations in Gaussian via an integral equation formalism polarizable continuum model (*22*–*39*) with ε = 9.9949, specifying SCRF=(Solvent=o-DiChloroBenzene), or a posteriori for energies via the COSMO-RS scheme. For the calculation with one [NEt_3_Me]^+^ cation, ONIOM calculations with two layers were performed. The first layer included the anionic species Cl^−^, [Cl_3_]^−^, the CO[Cl_3_]^−^ encounter complex, or the [C(O)Cl_2_][Cl]^−^ intermediate complex and was treated at the SCS-MP2/aug-cc-pVTZ level. The second layer included the cation and was treated at the M06-2X/cc-pVTZ level. The relative energies, characteristic for the reaction profile, were calculated in several steps. From the total electronic energies for all systems optimized in vacuum at the SCS-MP2 level or mixed ONIOM SCS-MP2:M06-2X level, ${\Delta E}_{\text{SCS}-\text{MP}2}^{0K}$ was calculated with respect to the energies of free CO and [Cl_3_]^−^. Zero-point energy corrections (Δε_ZPE_) as well as thermal correction $\left({\Delta \varepsilon }_{\text{therm}.}^{298.15K}\right)$ and entropic contributions (−*T*Δ*S*) were obtained from harmonic normal mode analyses. To incorporate higher-order electron correlation effects, a correction term Δε_CCSD(T) − F12_ was calculated from the difference of the SCS-MP2 and CCSD(T)-F12 energies of the anionic systems in vacuum and without any counterion. Correction terms to the Gibbs free energies of reactions in solution under standard conditions (Δ*G*_COSMO − RS_;298.15 K, 0.1 MPa) were obtained using the COSMO-RS solvation model (*40*–*43*). To this end, additional single-point calculations at the vacuum and polarizable continuum model (PCM) optimized structures were carried out using the TURBOMOLE program version 7.5.0 (*44*–*46*). These calculations were performed at the DFT-BP86 (*47*, *48*) level of theory in conjunction with def2-TZVPD (*49*) basis sets for all atoms, the multipole-accelerated resolution-of-identity approximation, and the refined COSMO cavity construction algorithm (keyword $cosmo_isorad) (*50*–*54*). Subsequent COSMO-RS computations used the COSMOtherm program version C30_1201 and a BP-TZVPD-FINE level parameterization (BP_TZVPD_FINE_HB2012_C30_1201). For an in-depth analysis of the reaction profile in terms of its thermochemistry, different thermochemical quantities were evaluated: the pure electronic energy Δ*E*^0*K*^, the enthalpy at 298.15 K with zero-point energy corrections added, Δ*H*^298.15*K*^, the Gibbs free energy in vacuum, Δ*G*^298.15*K*^, and the Gibbs free energy in *o*DCB solvent, Δ*G*^298.15*K*, *oDCB*^. All consecutive steps of the reaction of [Cl_3_]^−^ with CO were modeled with and without inclusion of the counter-cation [NEt_3_Me]^+^.

## Supplementary Materials

### This PDF file includes:

Electronic Supporting Information Supplementary Text Figs. S1 to S27 Tables S1 to S4 References
