# Supplementary material for: Novel synthetic pathway for the production of phosgene
Source: Sci Adv. 2021 Sep 29;7(40):eabj5186. doi: 10.1126/sciadv.abj5186 (PMC8480918; doi:10.1126/sciadv.abj5186)
Supplement: Supplementary file 1 — Electronic Supporting Information Supplementary Text Figs. S1 to S27 Tables S1 to S4 References [file sciadv.abj5186_sm.pdf]

Science Advances
AAAS

Supplementary Materials for

Novel synthetic pathway for the production of phosgene

Patrick Voßnacker, Alisa Wüst, Thomas Keilhack, Carsten Müller, Simon Steinhauer, Helmut Beckers, Sivathmeehan Yogendra, Yuliya Schiesser, Rainer Weber, Marc Reimann, Robert Müller, Martin Kaupp, Sebastian Riedel*

*Corresponding author. Email: s.riedel@fu-berlin.de

Published 29 September 2021, *Sci. Adv.* **7**, eabj5186 (2021)
DOI: 10.1126/sciadv.abj5186

This PDF file includes:

Electronic Supporting Information
Supplementary Text
Figs. S1 to S27
Tables S1 to S4
Movie S1
References

Experimental Setup

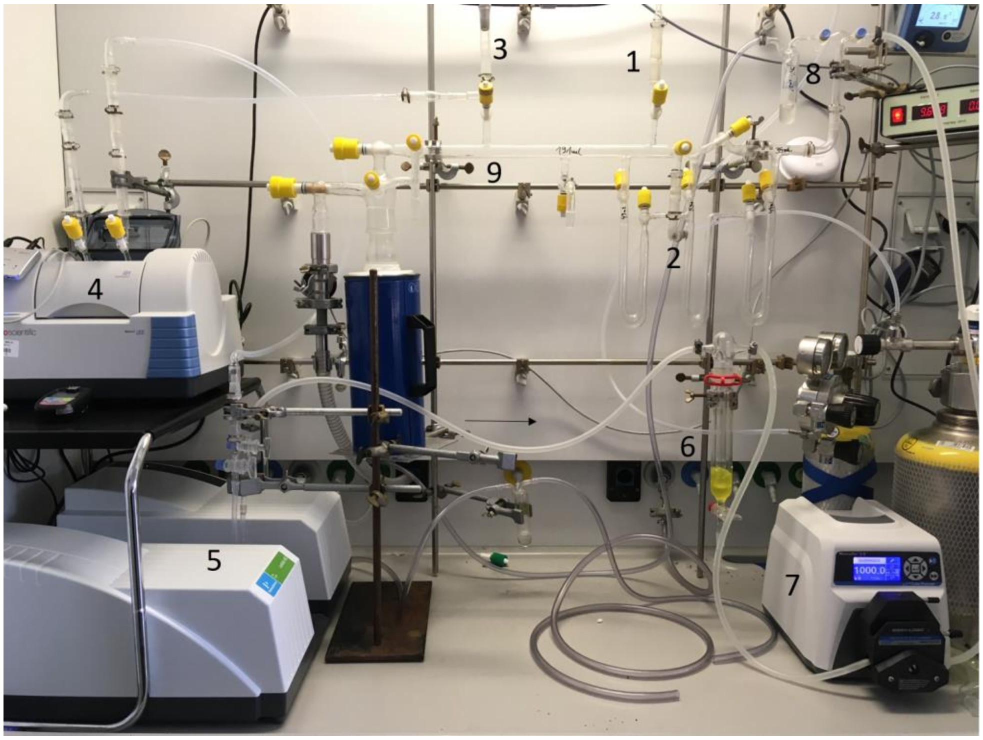

1 CO inlet

2 Cl_2 inlet

3 Pressure sensor

4 IR spectrometer

5 UV/Vis spectrometer

6 Gas wash bottle with diffusor und stopcock

7 Peristaltic pump

8 Bubbler with stopcock

9 Vacuum line

Fig. S1. Experimental setup for the investigation of the reaction of Cl_2 with CO.
Photo Credit: Patrick Voßnacker, FU Berlin

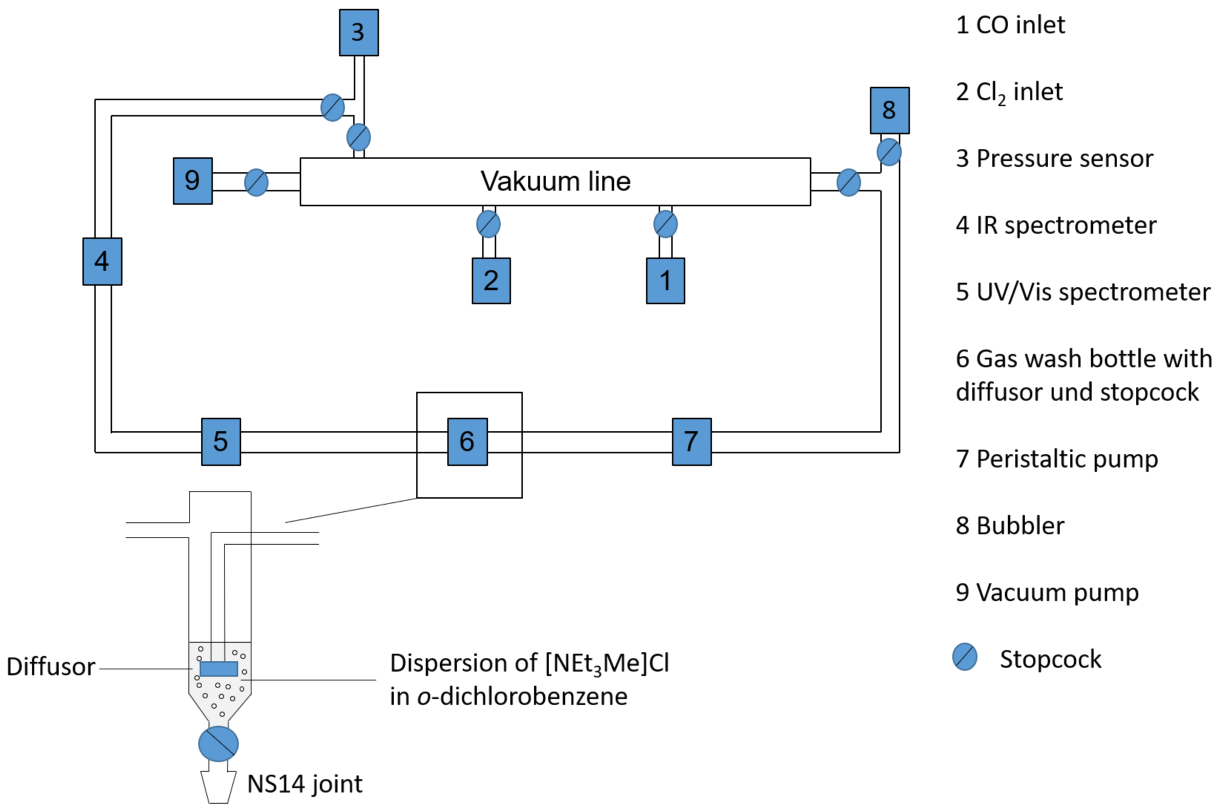

Fig. S2. Schematic representation of the experimental setup for the investigation of the reaction of Cl_2 with CO.

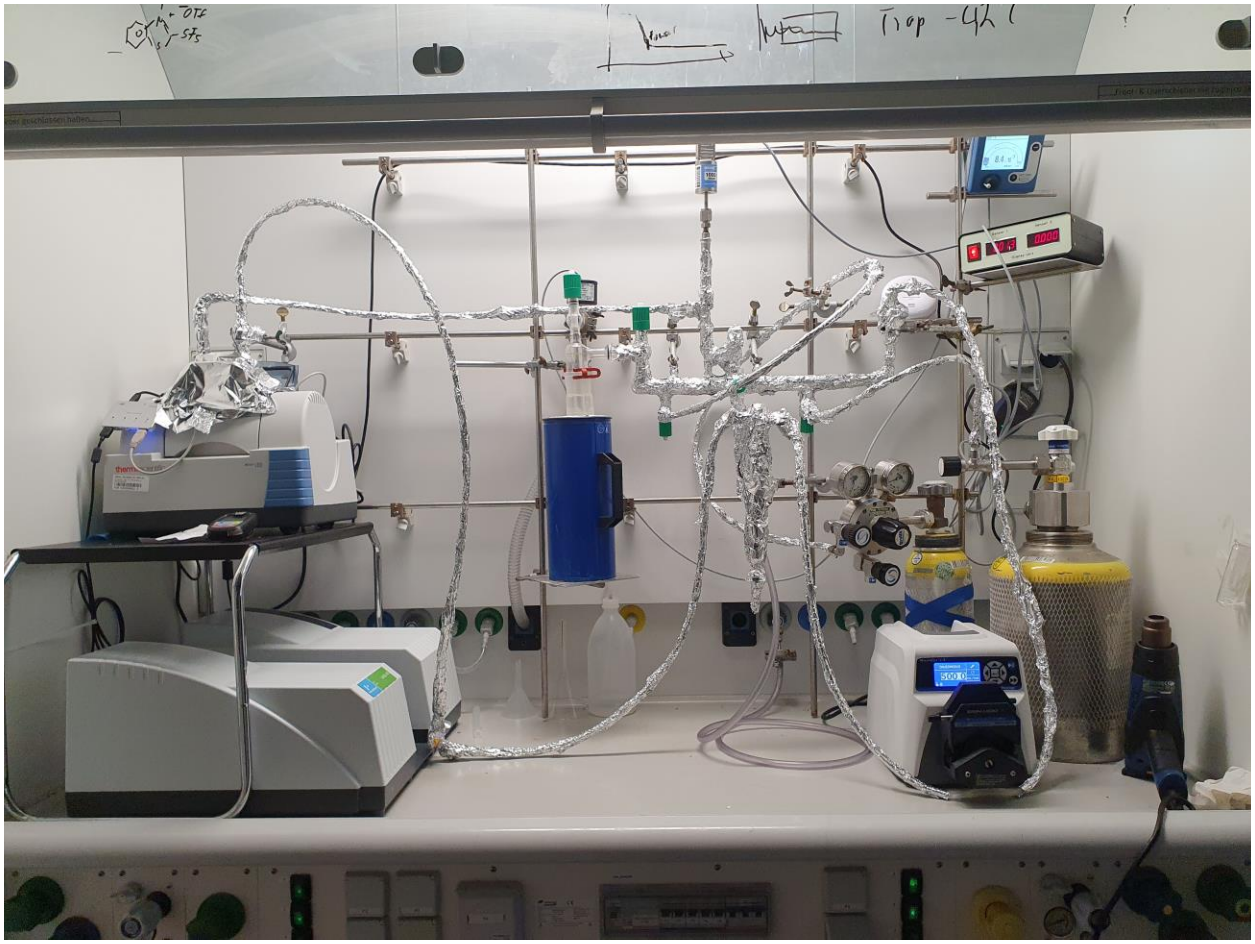

Fig. S3. Experimental setup for the investigation of the dark reaction of Cl_2 with CO. Photo Credit: Patrick Voßnacker, FU Berlin

Detailed Quantum-Chemical Results

A first method assessment, summarized in Table S1, showed that SCS-MP2 results deviate by less than 5% from high-level single-point CCSD(T)//SCS-MP2 relative energies for the intermediate complex ($[IC]^-$) with respect to free and separated CO and $[Cl_3]^-$, and for the overall electronic reaction energy (ΔE). MP2 single-point calculations, on the other hand, overestimate the formation energy of the $[IC]^-$ and the reaction energy of the products by about 10%. DFT results with several different functionals vary appreciably. M06-2X agrees best with the SCS-MP2 results and was hence applied subsequently in the ONIOM calculations with $[NEt_3Me]^+$. Addition of D3 dispersion corrections(*55*) was considered for B3LYP, M06, and M06-2X but had no appreciable impact on any value. Neglecting solvent effects completely at SCS-MP2 level has a dramatic destabilizing effect, reducing the relative electronic energy of the IC to −60.6 kJ/mol and the reaction energy to −16.0 kJ/mol. The corresponding Gibbs free energies at SCS-MP2 level (including solvent effects) are −72.8 and −81.2 kJ/mol, respectively. This means, the character of the final Cl^--abstraction step is endothermic but becomes exergonic once entropic contributions are considered. Consequently, we will focus the main discussion in the following on free energies in oDCB but provide also electronic energies and enthalpies for comparison.

Table S1. Calculated relative electronic energies (ΔE^{0K}) for the intermediate complex $[COCl_2][Cl^-]$ (**$[IC]^-$**) and the **total reaction** energy for the formation of $C(O)Cl_2$ and Cl^-. Energies are given with respect to free separated CO and $[Cl_3]^-$ and were calculated at different levels of theory. All energies (in kJ/mol) were calculated from full structure optimizations at the indicated levels, except for those at MP2 and CCSD(T) levels, which were obtained from single point calculations at SCS-MP2 optimized structures. For B3LYP, M06 and M06-2X, values in parenthesis were calculated including Grimme's D3 dispersion correction. Implicit solvent effects by *ortho*-dichlorobenzene were considered through the use of a polarizable continuum model (PCM).

	**SCS-MP2**	**$_{sp}$MP2**	**$_{sp}$CCSD(T)**	**B3LYP-D3**
**$[IC]^-$**	–99.8	–112.8	–97.4	–76.4(–81.2)
**total reaction**	–94.0	–104.9	–90.3	–74.0(–75.7)
	**M06-D3**	**M06-2X-D3**	**PBE0**	
**$[IC]^-$**	–106.3(–106.4)	–101.3(–101.5)	–121.4	
**total reaction**	–100.5(–100.3)	–93.8 (–93.7)	–116.5	

Table S2 and Fig. S4 show characteristic energies and free energies along the reaction path, calculated with different models. Considering only energies or enthalpies ($\Delta E^{0K}_{SCS-MP2}$ or $\Delta H^{298.15K}_{CCSD(T)-F12}$), CO and $[Cl_3]^-$ form a weakly bound encounter complex ($[EC]^-$) at a C-Cl distance of about 3.4 Å, which is, however, not stable on the free energy surface at room temperature ($\Delta G^{298.15K,oDCB}_{CCSD(T)-F12}$). When CO and $[Cl_3]^-$ get closer, one Cl-Cl bond is cleaved, forming a transition state ($[TS]^-$) with a relative free energy ($\Delta G^{298.15K,oDCB}_{CCSD(T)-F12}$) of +57 kJ/mol (omitting the counter-ion) or +78 kJ/mol (including one explicit cation). From there, energies would suggest formation of a stable $[COCl_2][Cl]^-$ intermediate complex ($[IC]^-$), with a structure very similar to that of the products, namely one chloride ion weakly bound to a phosgene molecule. In the phosgene moiety the C-O and C-Cl distances deviate by less than 0.012 Å from the equilibrium structure of isolated $COCl_2$. However, due to entropic reasons, this $[IC]^-$ dissociates without any free-energy barrier into the final products $COCl_2$ and $[Cl]^-$, and in total, the reaction is favorable by about 60 kJ/mol ($\Delta G^{298.15K,oDCB}_{CCSD(T)-F12}$).

Both the encounter complex $[EC]^-$ and the intermediate complex $[IC]^-$ are stable on the potential-energy surface, even after adding ZPE. But they are disfavored entropically and by solvent effects and do not exist on the free-energy surface in solution.

Table S2. Calculated characteristic energies and free energies along the reaction path. Relative energies (in kJ/mol) with respect to isolated CO and $[Cl_3]^-$ of the $[CO][Cl_3]^-$ encounter complex (**$[EC]^-$**), the transition state (**$[TS]^-$**), the intermediate $[COCl_2][Cl]^-$ complex (**$[IC]^-$**), and the overall **total reaction** energy are gvien. Where indicated, complexes of the anions $[EC]^-$, $[TS]^-$, $[IC]^-$, $[Cl_3]^-$ and Cl^- with the $[NEt_3Me]^+$ cation were modeled. All calculations were performed for isolated anions/ion pairs; solvent effects were included a posteriori. The energy contributions are the total electronic energies at SCS-MP2 level ($\boldsymbol{\Delta E^{0K}_{SCS-MP2}}$); the correction for higher order electron correlation effects at CCSD(T)-F12 level ($\Delta\boldsymbol{\varepsilon_{CCSD(T)-F12}}$); the zero point energy correction ($\Delta\boldsymbol{\varepsilon_{ZPE}}$); thermal corrections for 298.15 K ($\boldsymbol{\Delta\varepsilon^{298.15K}_{therm.}}$); the enthalpy at 298.15 K ($\boldsymbol{\Delta H^{298.15K}_{CCSD(T)-F12}} = \boldsymbol{\Delta E^{0K}_{SCS-MP2}} + \Delta\boldsymbol{\varepsilon_{CCSD(T)-F12}} + \Delta\boldsymbol{\varepsilon_{ZPE}} + \boldsymbol{\Delta\varepsilon^{298.15K}_{therm.}}$); entropic contributions at 298.15 K ($\boldsymbol{-T\Delta S}$); the free energy of solvation contribution ($\Delta\boldsymbol{G_{COSMO-RS}}$), and the resulting Gibbs free energy for 298.15 K ($\boldsymbol{\Delta G^{298.15K,oDCB}_{CCSD(T)-F12}} = \boldsymbol{\Delta H^{298.15K}_{CCSD(T)-F12}} + \Delta\boldsymbol{G_{COSMO-RS}}$) when summing up all terms.

	$\boldsymbol{\Delta E^{0K}_{SCS-MP2}}$	$\Delta\boldsymbol{\varepsilon_{CCSD(T)-F12}}$	$\Delta\boldsymbol{\varepsilon_{ZPE}}$	$\boldsymbol{\Delta\varepsilon^{298.15K}_{therm.}}$
**$[EC]^-$**	**−11.3**	+1.0	+0.5	−3.0
**$[TS]^-$**	**+67.3**	−3.3	+2.4	−2.1
**$[IC]^-$**	**−68.5**	+8.6	+10.3	−4.0
**total reaction**	**−21.8**	+9.5	+9.3	−3.9
	$\boldsymbol{\Delta H^{298.15K}_{CCSD(T)-F12}}$	$\boldsymbol{-T\Delta S}$	$\Delta\boldsymbol{G_{COSMO-RS}}$	$\boldsymbol{\Delta G^{298.15K,oDCB}_{CCSD(T)-F12}}$
**$[EC]^-$**	**−12.8**	+29.5	−14.7	**+2.0**
**$[TS]^-$**	**+64.3**	+28.8	−36.2	**+56.9**
**$[IC]^-$**	**−53.6**	+34.9	−33.8	**−52.5**
**total reaction**	**−6.9**	+8.6	−61.1	**−59.4**
	$\boldsymbol{\Delta E^{0K}_{SCS-MP2}}$	$\Delta\boldsymbol{\varepsilon_{CCSD(T)-F12}}$	$\Delta\boldsymbol{\varepsilon_{ZPE}}$	$\boldsymbol{\Delta\varepsilon^{298.15K}_{therm.}}$
**$[EC]^-[NEt_3Me]^+$**	**−9.7**	+0.6	+1.0	−1.8
**$[TS]^-[NEt_3Me]^+$**	**+37.1**	−4.1	+3.4	−3.7
**$[IC]^-[NEt_3Me]^+$**	**−95.1**	+8.8	+9.5	−2.1
**total reaction**	**−61.3**	+9.3	+7.1	−5.5
	$\boldsymbol{\Delta H^{298.15K}_{CCSD(T)-F12}}$	$\boldsymbol{-T\Delta S}$	$\Delta\boldsymbol{G_{COSMO-RS}}$	$\boldsymbol{\Delta G^{298.15K,oDCB}_{CCSD(T)-F12}}$
**$[EC]^-[NEt_3Me]^+$**	**−9.9**	+37.5	−8.0	**+19.6**
**$[TS]^-[NEt_3Me]^+$**	**+32.7**	+44.8	+0.1	**+77.6**
**$[IC]^-[NEt_3Me]^+$**	**−78.9**	+24.1	−12.0	**−56.8**
**total reaction**	**−50.4**	−2.2	−10.4	**−63.0**

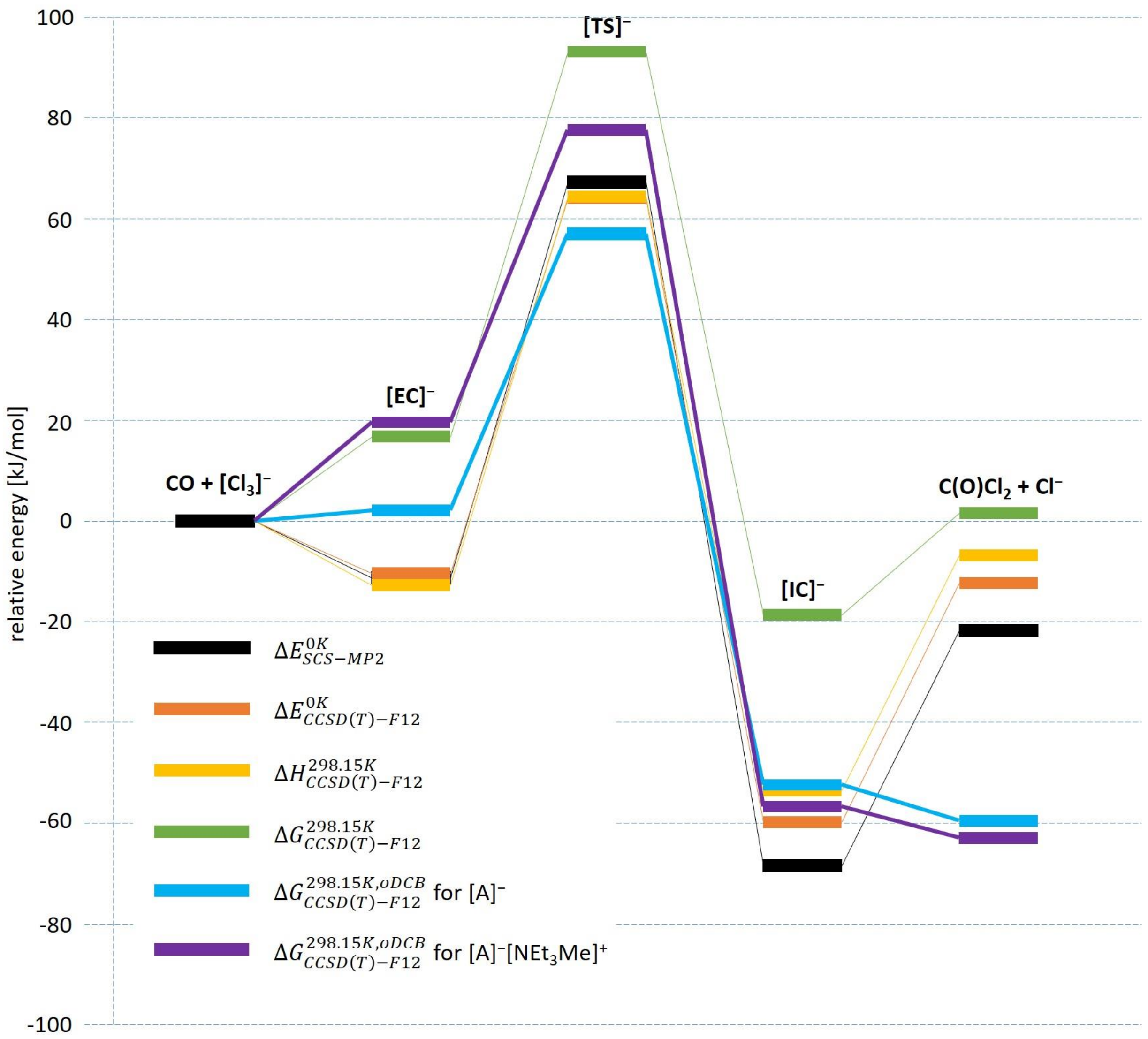

Fig. S4. Calculated reaction path for the reaction of CO and $[Cl_3]^-$. Relative energies and free energies (in kJ/mol) with respect to isolated CO and $[Cl_3]^-$ of the $[CO][Cl_3]^-$ encounter complex (**[EC]**$^-$), the transition state (**[TS]**$^-$), the intermediate $[COCl_2][Cl]^-$ complex (**[IC]**$^-$), and the products $C(O)Cl_2$ and Cl^-. All calculations were performed for isolated anions/ion pairs; solvent effects were included a posteriori. Reaction profiles are derived from the total electronic energy at SCS-MP2 level ($\boldsymbol{\Delta E^{0K}_{SCS-MP2}}$; black), including higher-order electron correlation effects at CCSD(T)-F12 level ($\boldsymbol{\Delta E^{0K}_{CCSD(T)-F12}} = \boldsymbol{\Delta E^{0K}_{SCS-MP2}} + \Delta\varepsilon_{CCSD(T)-F12}$; orange), including zero point energy correction and thermal corrections for 298.15 K ($\boldsymbol{\Delta H^{298.15K}_{CCSD(T)-F12}} = \boldsymbol{\Delta E^{0K}_{CCSD(T)-F12}} + \Delta\varepsilon_{ZPE} + \boldsymbol{\Delta\varepsilon^{298.15K}_{therm.}}$; yellow), including entropic contributions to the Gibbs free energy for 298.15 K ($\boldsymbol{\Delta G^{0K}_{CCSD(T)-F12}} = \boldsymbol{\Delta H^{298.15K}_{CCSD(T)-F12}} - \boldsymbol{T}\Delta\boldsymbol{S}$; green), including solvent effects for orthodichlorobenzene calculated with COSMO-RS but no cations ($\boldsymbol{\Delta G^{298.15K,oDCB}_{CCSD(T)-F12}}$ for $[A]^-$; blue), and including one explicit $[NEt_3Me]^+$ cation for each anion ($\boldsymbol{\Delta G^{298.15K,oDCB}_{CCSD(T)-F12}}$ for $[A]^-[NEt_3Me]^+$; violet).

The cation and the solvent complement each other in stabilizing the anionic species. That is, solvent effects are diminished when a cation is included in the computations, as one might expect. The cation reduces the electronic contribution to the barrier by about 30 kJ mol^{-1} but at the same time increases the entropic barrier by about 15 kJ mol^{-1} and the solvent contribution by about 24

kJ mol^{-1}. A moderate overall increase of the free-energy barrier by about 20 kJ mol^{-1} results. We note, however, that these reaction steps likely do not occur in the organic phase but in the IL phase or at the interface between the two phases (see below). In that sense, the employed solvent model and the single counter-ion only approximate the true environmental effects.

The distribution of the involved stable species between the oDCB and the IL phase was investigated by COSMO-RS calculations of the equilibrium mole fractions (Table S3). While the gaseous compounds (CO, Cl_2, $COCl_2$) have a decent to clear preference for the organic phase, all ionic species, including the chloride ion, prefer almost exclusively the IL phase. Even contact ion pairs of $[NEt_3Me][Cl_3]$ clearly prefer the IL phase. Since the $[IC]^-$ intermediate shows a very clear preference for the IL phase as well, the reaction most likely takes place in the IL or at the interface between both phases, while gaseous CO and $COCl_2$ move to or from the IL through the organic phase. This is in agreement with the experimental finding that the reaction takes place in the pure ionic liquid as well.

Table S3. Equilibrium mole fractions of various compounds in the organic (phase 1) and the IL phase (phase 2), calculated at the COSMO-RS level, as well as the ratio of the two molar fractions. The equilibrium was calculated starting from a pure oDCB phase and an IL phase containing equal parts of $[NEt_3Me]^+$ and $[Cl_3]^-$ (x_0=0.49455984) and all other compounds, respectively (x_0=0.00098912).

Compound	x in phase 1 (oDCB)	x in phase 2 (IL)	x_1/x_2
**CO**	0.00089679	0.00018638	4.8
**Cl_2**	0.00093889	0.00012016	7.8
**$COCl_2$**	0.00096326	0.00008484	11.4
**Cl^-**	0.00000001	0.00073359	$1.4 \cdot 10^{-5}$
**$[Cl_3]^-$**	0.00019333	0.47089775	$4.1 \cdot 10^{-4}$
**$[NEt_3Me]^+$**	0.00019421	0.47283414	$4.1 \cdot 10^{-4}$
**$[NEt_3Me]^+$ $[Cl_3]^-$**	0.00012376	0.00130763	$9.5 \cdot 10^{-2}$
**IC**	0.00000087	0.00120280	$7.2 \cdot 10^{-4}$
***o*DCB**	0.99668887	0.05263270	18.9

Molecular Structures

Cartesian coordinates (in Ångström) from optimizations at SCS-MP2/aug-cc-pVTZ level for all systems without $[NEt_3Me]^+$ or with ONIOM (SCS-MP2/aug-cc-pVTZ:M06-2X/cc-pVTZ) for all systems with $[NEt_3Me]^+$. No solvent models were applied during the optimizations. Each line refers to one atom, containing: a continuous number, the atomic number and the x, y, and z coordinate in an arbitrary three-dimensional Cartesian coordinate system.

carbon monoxide --- CO

1	6	0.000000	0.000000	0.000000
2	8	1.136400	0.000000	0.000000

trichloride --- $[Cl_3]^-$

1	17	0.000000	0.000000	0.000000
2	17	2.317300	0.000000	0.000000
3	17	-2.317300	0.000000	0.000000

phosgene --- $C(O)Cl_2$

1	17	1.447090	-0.483050	-0.000002
2	17	-1.447090	-0.483050	-0.000002
3	6	-0.000000	0.496780	0.000023
4	8	0.000000	1.680375	-0.000008

encounter complex ($[EC]^-$) --- $CO[Cl_3]^-$

1	6	1.686805	2.061250	0.656033
2	8	1.773912	2.215708	-0.467011
3	17	-2.597394	0.335838	-0.002855
4	17	-0.483791	-0.596151	-0.003088
5	17	1.651060	-1.509874	-0.005828

transition state ($[TS]^-$) --- $[ClO(C)Cl_2]^-$

1	6	1.237324	-1.039218	-0.000000
2	8	2.261997	-1.549988	-0.000000
3	17	-0.940758	2.941711	0.000000
4	17	-0.000000	1.078018	0.000000
5	17	-0.560414	-2.923539	-0.000000

intermediate complex ($[IC]^-$) --- $C(O)Cl_2[Cl]^-$

1	17	-0.453801	-2.450094	0.000000
2	17	0.000000	0.435316	0.000000
3	6	-1.186389	-0.802977	0.000000
4	8	-2.369686	-0.678641	0.000000
5	17	1.987673	2.617542	0.000000

cation-chloride-pair --- $[NEt_3Me]^+Cl^-$

1	7	0.828370	-0.001430	0.004410
2	6	2.310567	-0.007764	0.017571
3	1	2.676670	-0.321818	-0.955356
4	1	2.657979	-0.698760	0.780010
5	1	2.671099	0.992367	0.239271
6	6	0.308666	-1.373336	-0.390532
7	6	0.451447	-1.703856	-1.862555
8	1	1.464027	-1.580218	-2.247698
9	1	-0.240954	-1.121262	-2.465490
10	1	0.182066	-2.751679	-1.984362
11	1	0.858873	-2.083194	0.224156
12	1	-0.749858	-1.363844	-0.118664
13	6	0.323391	1.026045	-0.994239
14	6	0.452825	2.465889	-0.540635
15	1	1.458761	2.741932	-0.222942
16	1	-0.255388	2.692415	0.252518
17	1	0.195714	3.094491	-1.391564
18	1	0.890078	0.851180	-1.906697
19	1	-0.731940	0.783425	-1.140890
20	6	0.302489	0.349277	1.385770
21	6	0.413291	-0.764488	2.407172
22	1	-0.299978	-1.558773	2.200699
23	1	0.151509	-0.339521	3.374822
24	1	1.414309	-1.187044	2.496767
25	1	0.866426	1.225537	1.699764
26	1	-0.750160	0.599678	1.233668
27	17	-2.684346	0.004970	-0.013776

cation-trichloride-pair --- $[NEt_3Me]^+[Cl_3]^-$

1	7	1.856939	0.000276	-0.174425
2	6	3.285138	0.000570	-0.582528
3	1	3.771014	-0.885670	-0.186217
4	1	3.770808	0.886697	-0.185717
5	1	3.340061	0.000883	-1.668021
6	6	1.804613	-0.000041	1.333555
7	6	0.407493	-0.000186	1.911838
8	1	-0.163296	0.881507	1.620913
9	1	0.500623	-0.000510	2.997188
10	1	-0.163339	-0.881675	1.620375
11	1	2.365566	-0.877965	1.648299
12	1	2.365548	0.877763	1.648670
13	6	1.157793	1.214337	-0.759100
14	6	1.566320	2.547804	-0.174305
15	1	1.017410	3.309273	-0.725768
16	1	2.630654	2.761369	-0.268191
17	1	1.261040	2.642805	0.865848
18	1	1.362143	1.170174	-1.828378
19	1	0.092277	1.063894	-0.613535
20	6	1.158160	-1.213757	-0.759684
21	6	1.566524	-2.547317	-0.174991
22	1	2.631008	-2.760557	-0.267968
23	1	1.018300	-3.308733	-0.727206
24	1	1.260267	-2.642760	0.864837
25	1	0.092547	-1.063489	-0.614589
26	1	1.363060	-1.169333	-1.828845
27	17	-2.055815	2.302612	-0.099079
28	17	-2.195042	-0.000183	-0.149172
29	17	-2.054468	-2.303257	-0.098775

cation-encounter-complex-pair --- $[NEt_3Me]^+[EC]^-$

1	6	1.816408	1.025532	-1.123072
2	7	1.823485	0.033561	-0.004280
3	6	0.977803	0.561889	1.140960
4	6	1.345285	1.951596	1.617253
5	6	1.194809	-1.237828	-0.540549
6	6	1.015353	-2.345338	0.472978
7	6	3.223839	-0.203534	0.488392
8	6	4.148006	-0.884039	-0.502045
9	17	-1.886340	0.395388	-0.932461
10	17	-1.346852	2.642290	-0.585514
11	17	-2.200836	-1.846225	-1.160288
12	1	2.429812	1.878493	-0.847905
13	1	0.789403	1.347988	-1.289782
14	1	2.215136	0.550500	-2.013978
15	1	1.952347	-2.662952	0.932099
16	1	0.591912	-3.194481	-0.060652
17	1	0.297561	-2.074910	1.244234
18	1	0.230660	-0.945332	-0.946266
19	1	1.825773	-1.556319	-1.366835
20	1	4.200753	-0.352675	-1.450637
21	1	3.862536	-1.916699	-0.689910
22	1	5.149668	-0.891451	-0.075610
23	1	3.615570	0.772967	0.760947
24	1	3.135944	-0.793304	1.398612
25	1	1.169392	2.698631	0.847276
26	1	2.363583	2.024753	1.997153
27	1	0.669782	2.190182	2.437483
28	1	-0.047383	0.550752	0.779190
29	1	1.073363	-0.165028	1.943566
30	6	-2.084603	-0.804885	2.292375
31	8	-3.216642	-0.791388	2.360462

cation-transition state-pair --- $[NEt_3Me]^+[TS]^-$

1	6	-0.994615	-0.808380	-1.493237
2	7	0.068441	-0.415770	-0.519753
3	6	0.121861	1.099510	-0.418132
4	6	0.276936	1.829650	-1.736117
5	6	-0.339283	-0.965895	0.834967
6	6	0.681158	-0.764936	1.935334
7	6	1.407971	-0.935465	-0.953234
8	6	1.529155	-2.446556	-0.976242
9	17	-0.786860	2.625886	2.517084
10	17	-3.248863	0.962484	0.454727
11	17	1.372789	2.932226	2.476965
12	1	-0.676573	-0.532128	-2.494410
13	1	-1.910683	-0.289258	-1.209052
14	1	-1.144075	-1.881978	-1.435376
15	1	1.604317	-1.318124	1.762974
16	1	0.240329	-1.144066	2.856238
17	1	0.919449	0.282997	2.104905
18	1	-1.284888	-0.471186	1.062253
19	1	-0.539456	-2.024418	0.679963
20	1	0.810929	-2.908206	-1.650833
21	1	1.416221	-2.884793	0.013090
22	1	2.524932	-2.698817	-1.337003
23	1	1.584926	-0.528859	-1.945818
24	1	2.140610	-0.497954	-0.278044
25	1	-0.603398	1.723973	-2.366529
26	1	1.165521	1.543747	-2.297414
27	1	0.374528	2.887139	-1.494426
28	1	-0.817631	1.384253	0.052429
29	1	0.945098	1.332214	0.252712
30	6	-2.914772	2.355341	2.558150
31	8	-3.925002	2.555809	3.042568

cation-intermediate complex-pair --- $[NEt_3Me]^+[IC]^-$

1	6	-2.584915	-2.121630	-0.072786
2	7	-2.046890	-0.741211	-0.023410
3	6	-3.049250	0.183269	0.644590
4	6	-4.248097	0.542401	-0.209746
5	6	-1.769945	-0.241610	-1.430947
6	6	-0.544908	-0.845974	-2.087505
7	6	-0.751645	-0.714366	0.766831
8	6	-0.910406	-0.872015	2.264996
9	17	-0.784120	2.584984	0.079077
10	1	-2.738850	-2.482563	0.939762
11	1	-1.875851	-2.764257	-0.586090
12	1	-3.530225	-2.118421	-0.607412
13	1	-1.469038	-1.760870	2.558723
14	1	-1.364884	0.008371	2.712959
15	1	0.091952	-0.965606	2.681078
16	1	-0.135621	-1.511337	0.356089
17	1	-0.310376	0.258655	0.544706
18	1	-4.775562	-0.321582	-0.614610
19	1	-3.968693	1.211674	-1.019856
20	1	-4.947243	1.081676	0.427270
21	1	-3.357869	-0.327791	1.554521
22	1	-2.486681	1.087861	0.885151
23	1	0.371686	-0.488100	-1.623562
24	1	-0.536857	-0.509989	-3.123184
25	1	-0.541875	-1.936227	-2.097708
26	1	-2.668223	-0.463559	-2.003645
27	1	-1.637513	0.837809	-1.328595
28	17	2.263094	1.233042	-0.017966
29	6	3.539237	0.072422	-0.011595
30	17	2.905561	-1.587926	0.032101
31	8	4.699054	0.303255	-0.032419

Spectra

[NEt_3Me][Cl_3] + CO (bulk)

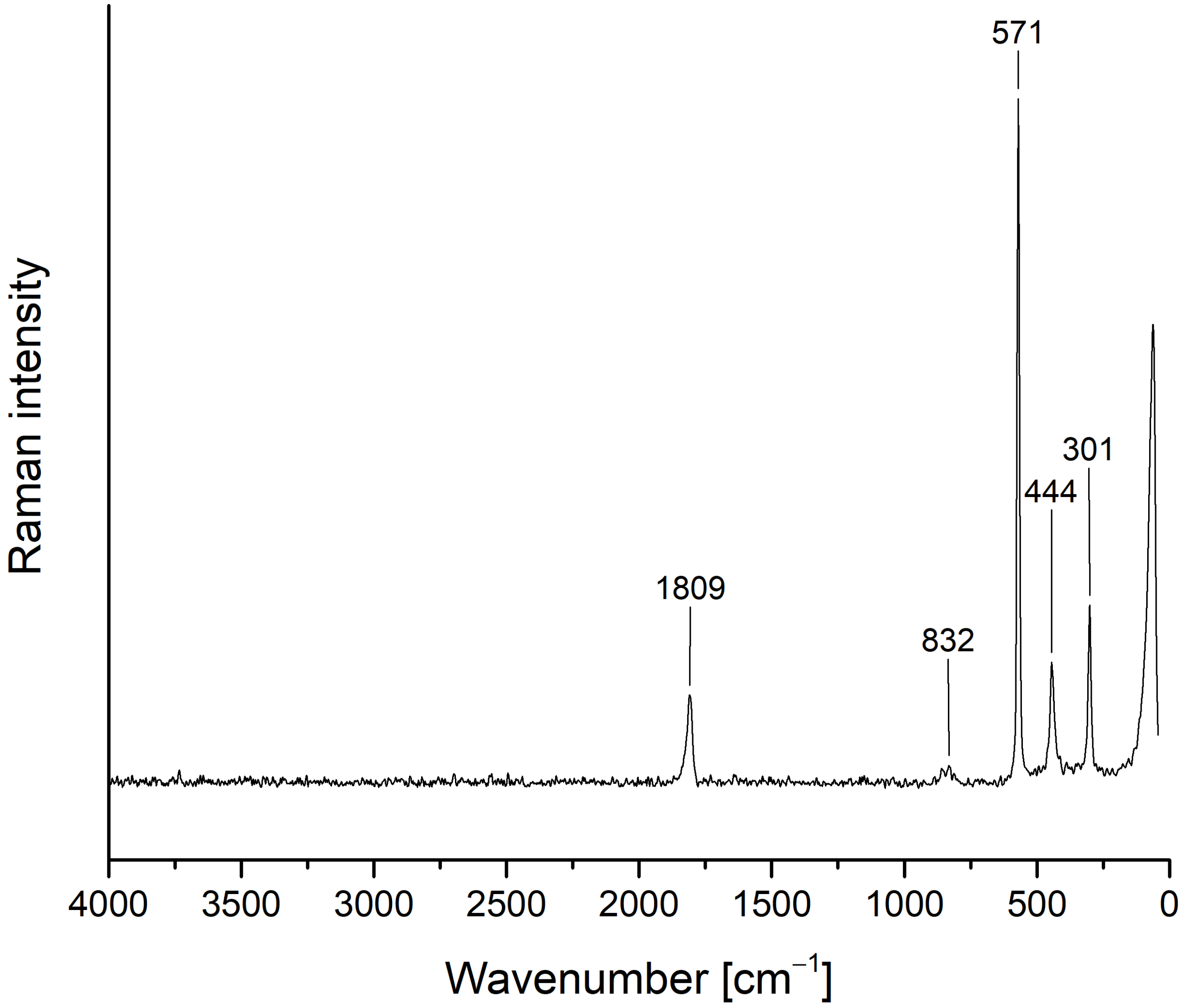

Fig. S5. Raman spectrum of purified $COCl_2$ obtained from the reaction of [NEt_3Me][Cl_3] + CO in *o*DCB.

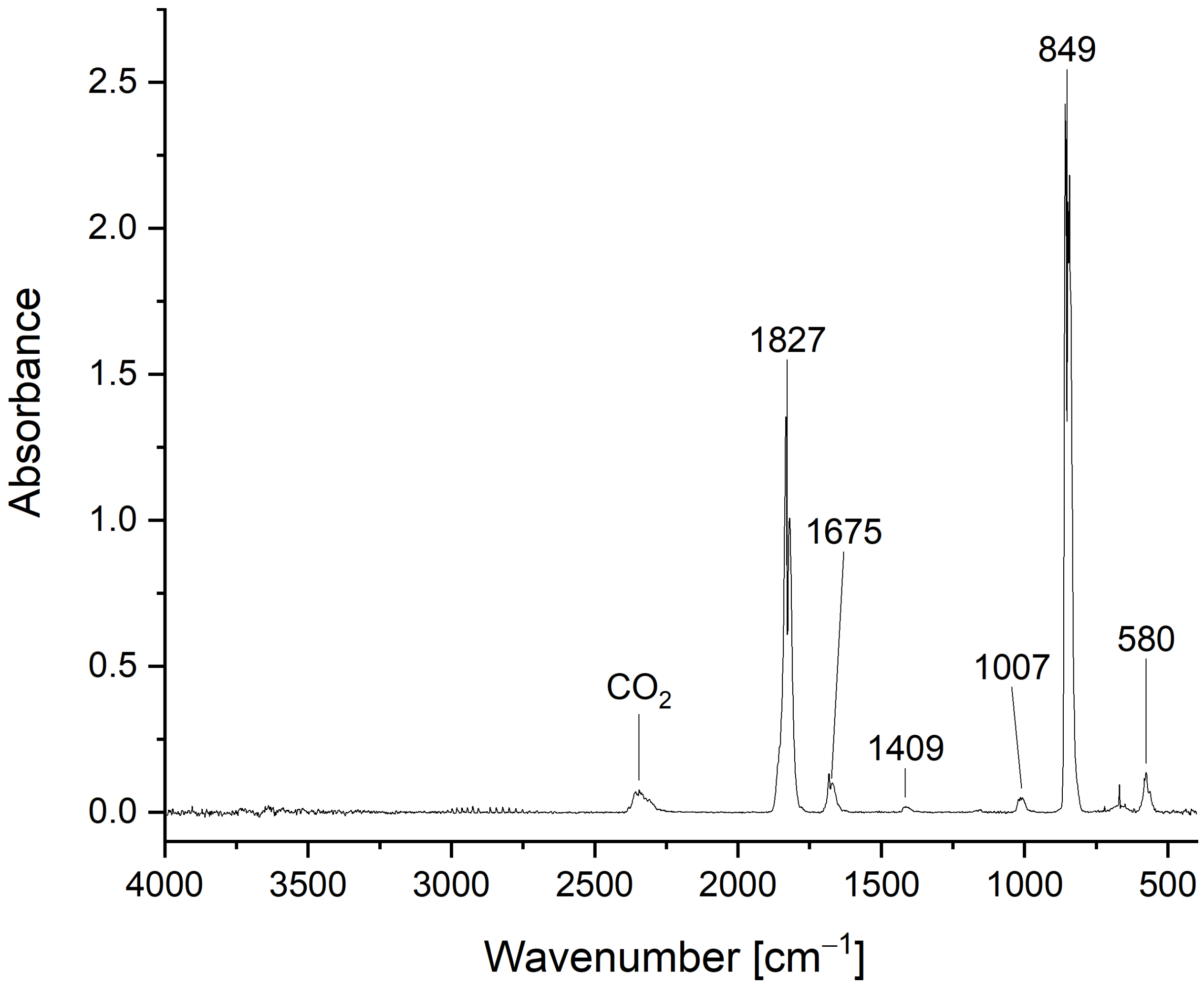

Fig. S6. Gas-phase IR spectrum of purified $COCl_2$ obtained from the reaction of $[NEt_3Me][Cl_3]$ + CO in *o*DCB.

3.5 mol% [NEt_3Me]Cl + Cl_2 + CO (bulk)

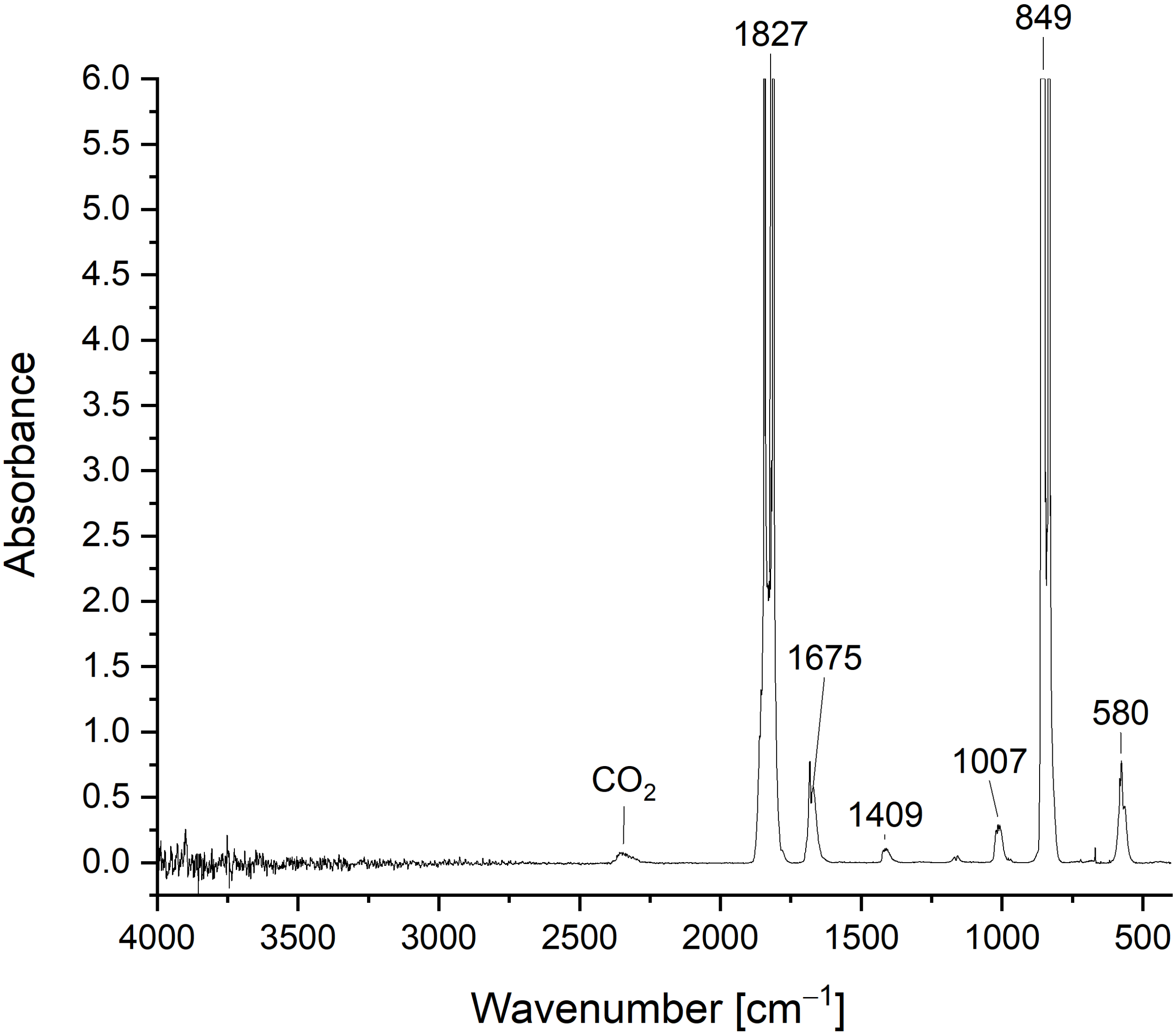

Fig. S7. Gas-phase IR spectrum of purified $COCl_2$ obtained from the reaction of Cl_2 + CO in *o*DCB catalyzed by 3.5 mol% [NEt_3Me]Cl.

[NEt$_3$Me][Cl(Cl$_2$)$_{1.45}$]

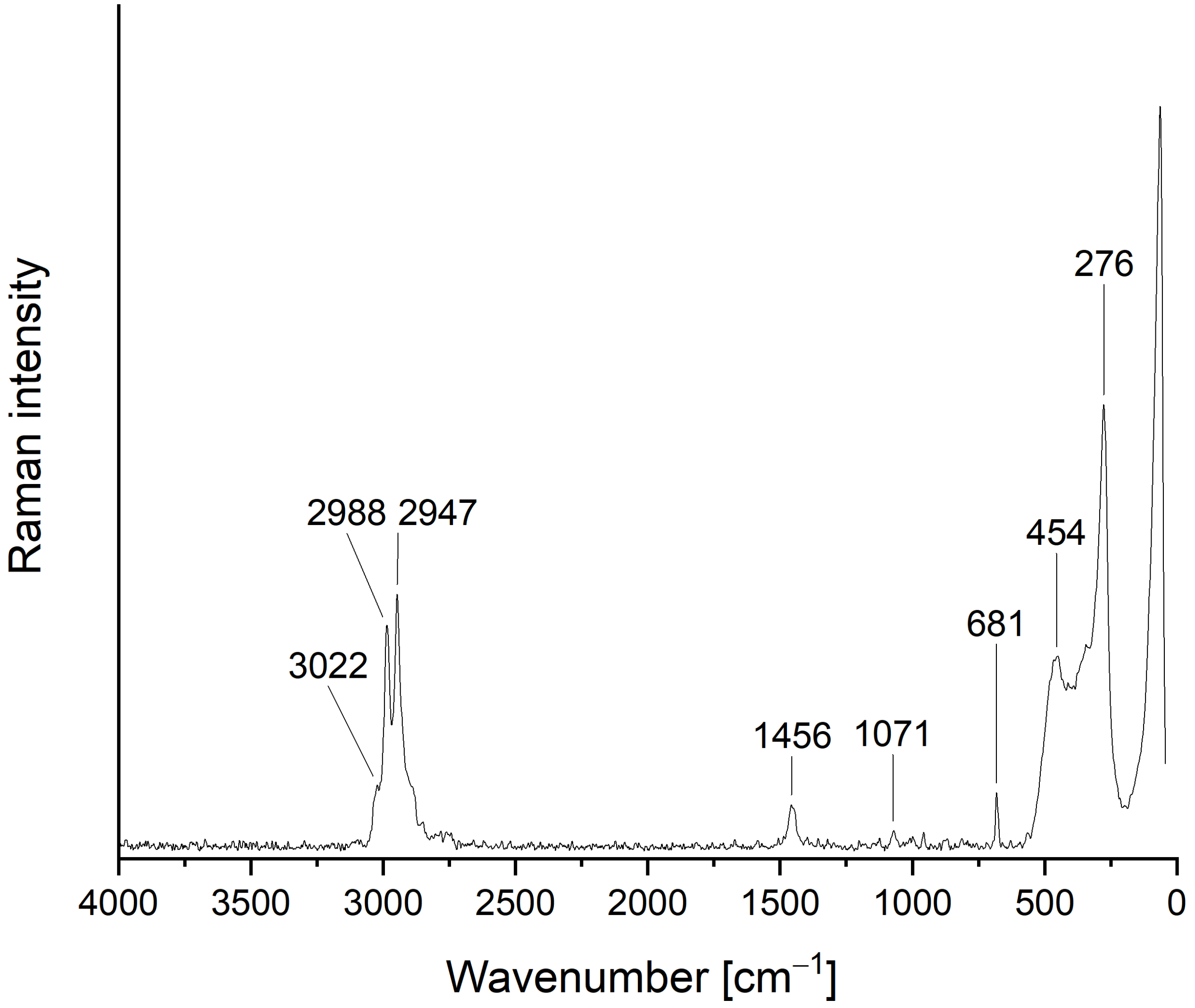

Fig. S8. Raman spectrum of [NEt$_3$Me][Cl(Cl$_2$)$_{1.45}$]

[NEt$_3$Me]Cl + CO + Cl$_2$ (flow)

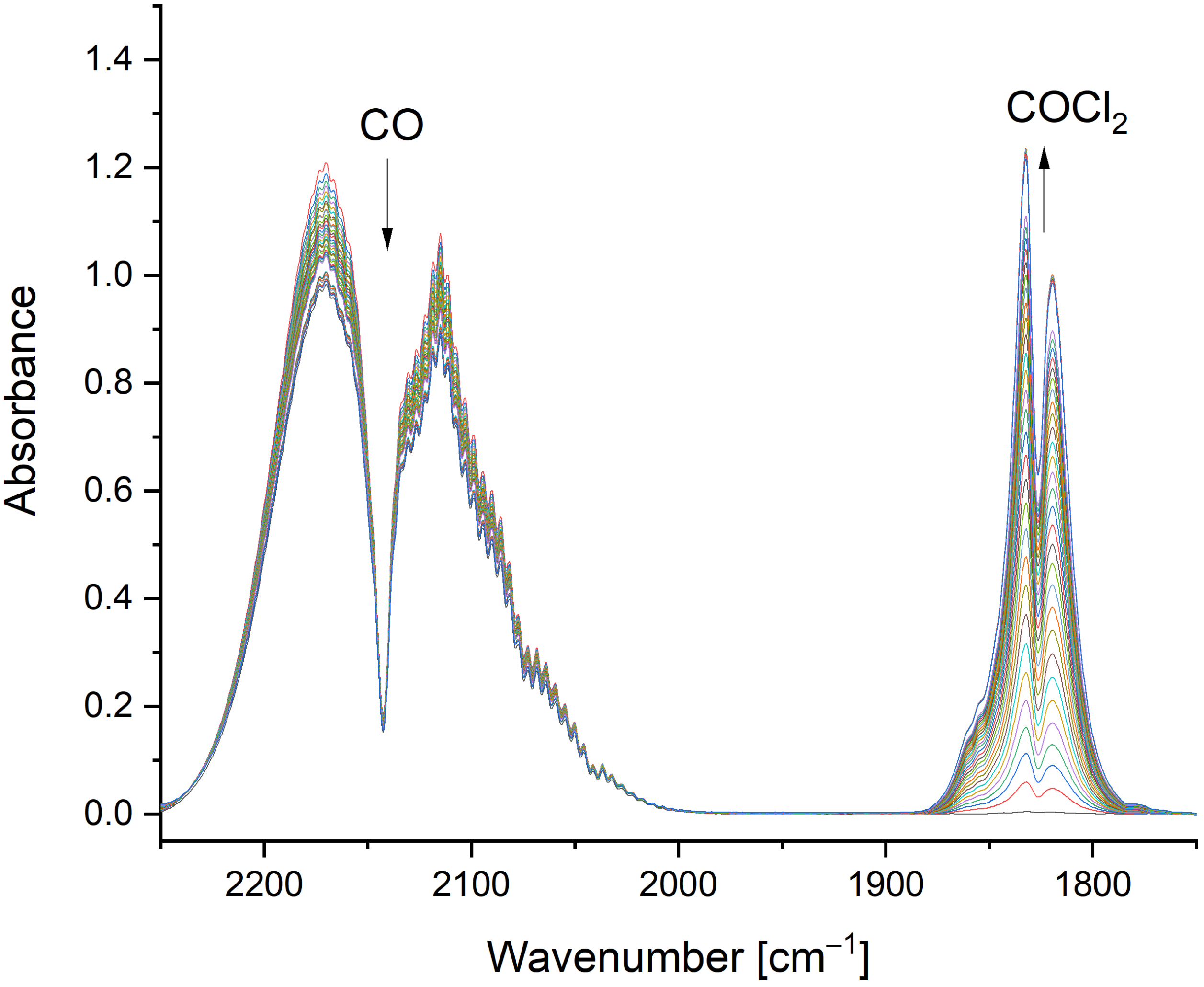

Fig. S9. Time-dependent gas-phase IR spectra of the gaseous constituents obtained from the reaction of [NEt$_3$Me]Cl + CO + Cl$_2$. Spectra were recorded after 0, 6, 11, 16, 21, 26, 31, 36, 41, 46, 51, 56, 61, 66, 71, 76, 81, 86, 91, 96, 101, 106, 111, 116, 121, 126, 131, 136, 141, 184, 191, 196, 201, 206, 211, 216, 221, 226 min.

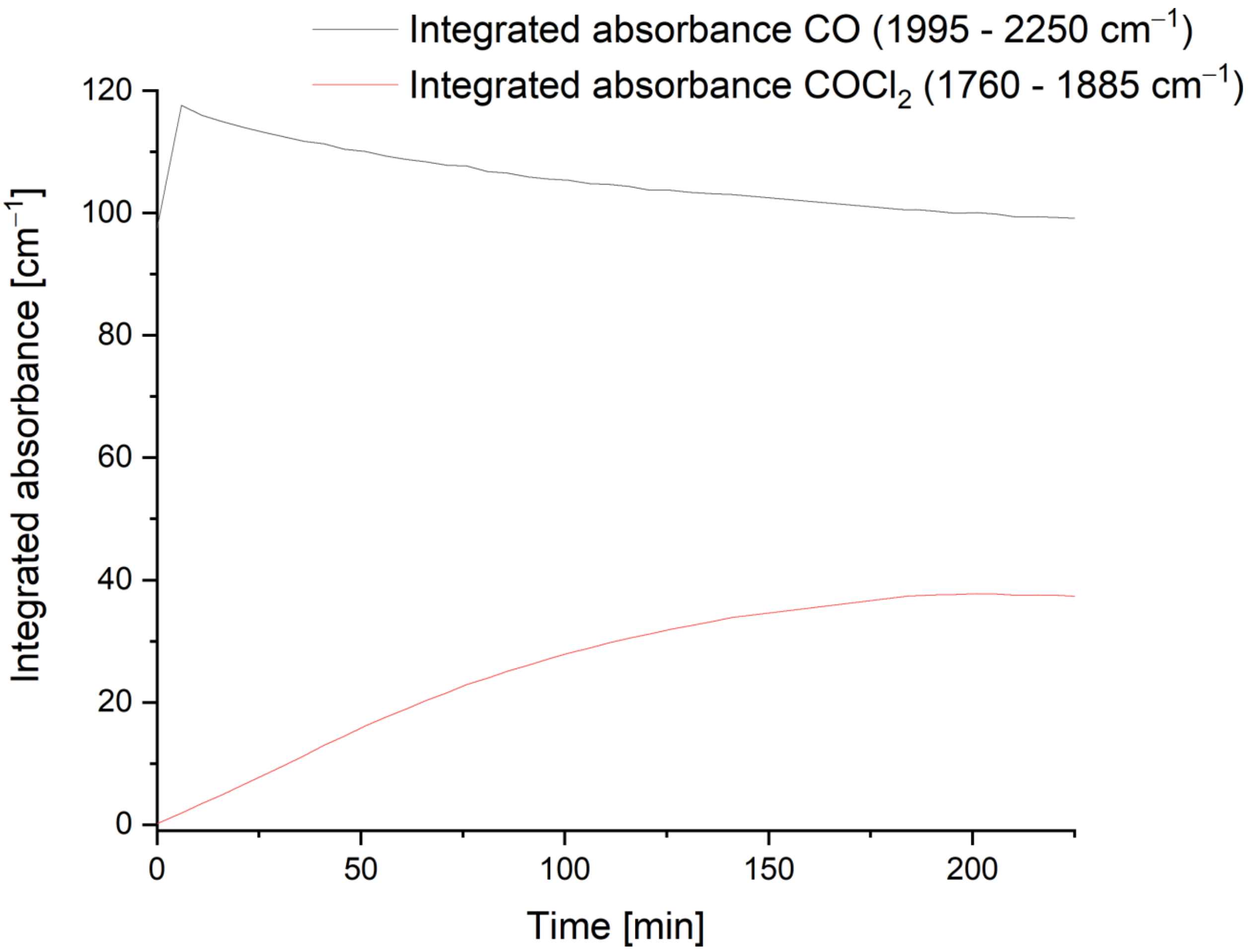

Fig. S10. Time-dependent integrated gas-phase IR absorbances for the CO stretching bands of CO (black line, 1995 – 2250 cm^{-1}) and $COCl_2$ (red line, 1760 – 1885 cm^{-1}) taken for the reaction of $[NEt_3Me]Cl$ + CO + Cl_2 at reaction times between 0 and 226 min.

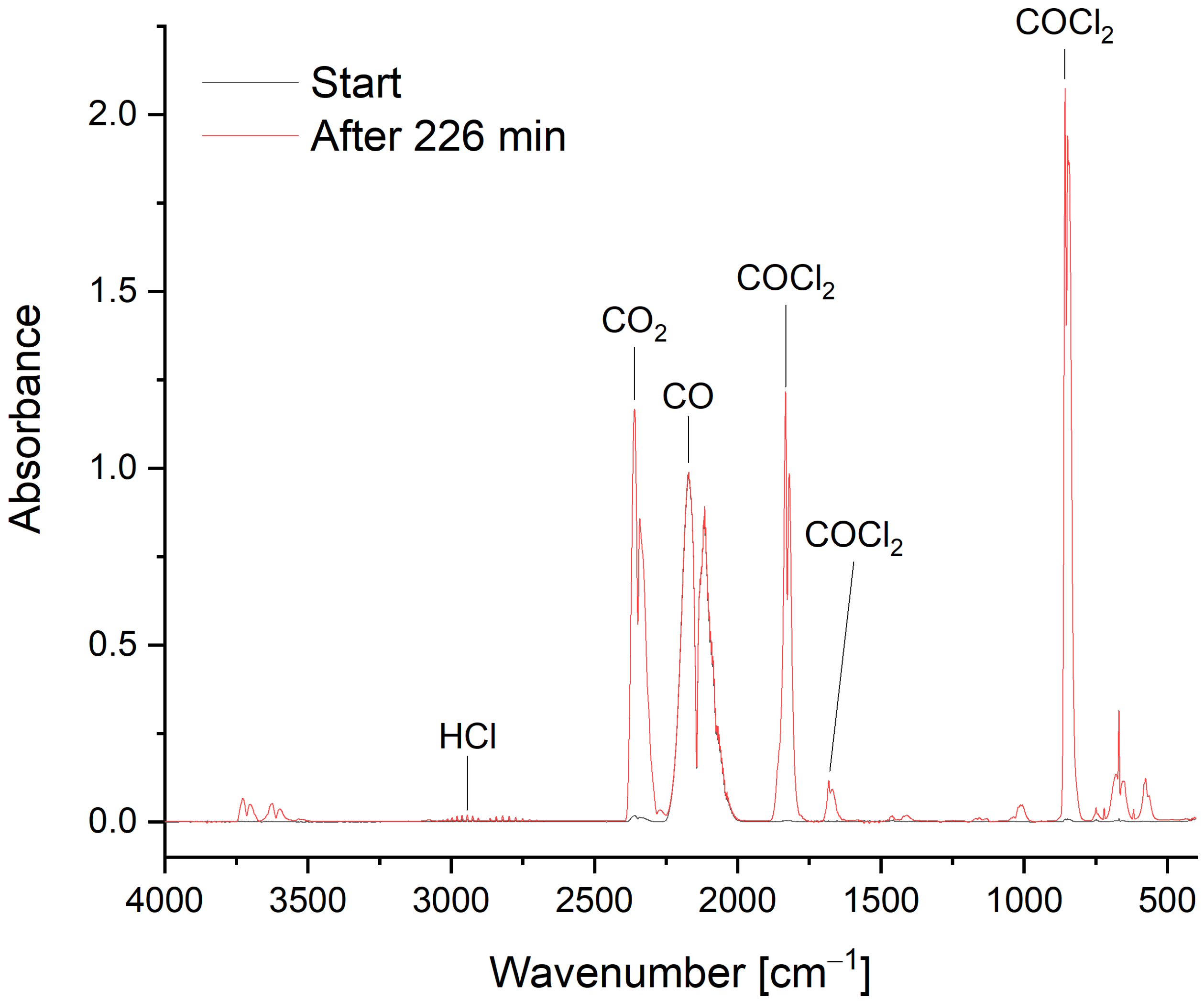

Fig. S11. Gas-phase IR spectra of the gaseous constituents obtained in the reaction of [NEt_3Me]Cl + CO + Cl_2 after a reaction time of 0 (black spectrum) and 226 min (red spectrum), respectively.

$[NEt_3Me][Cl_3]$ + CO (flow)

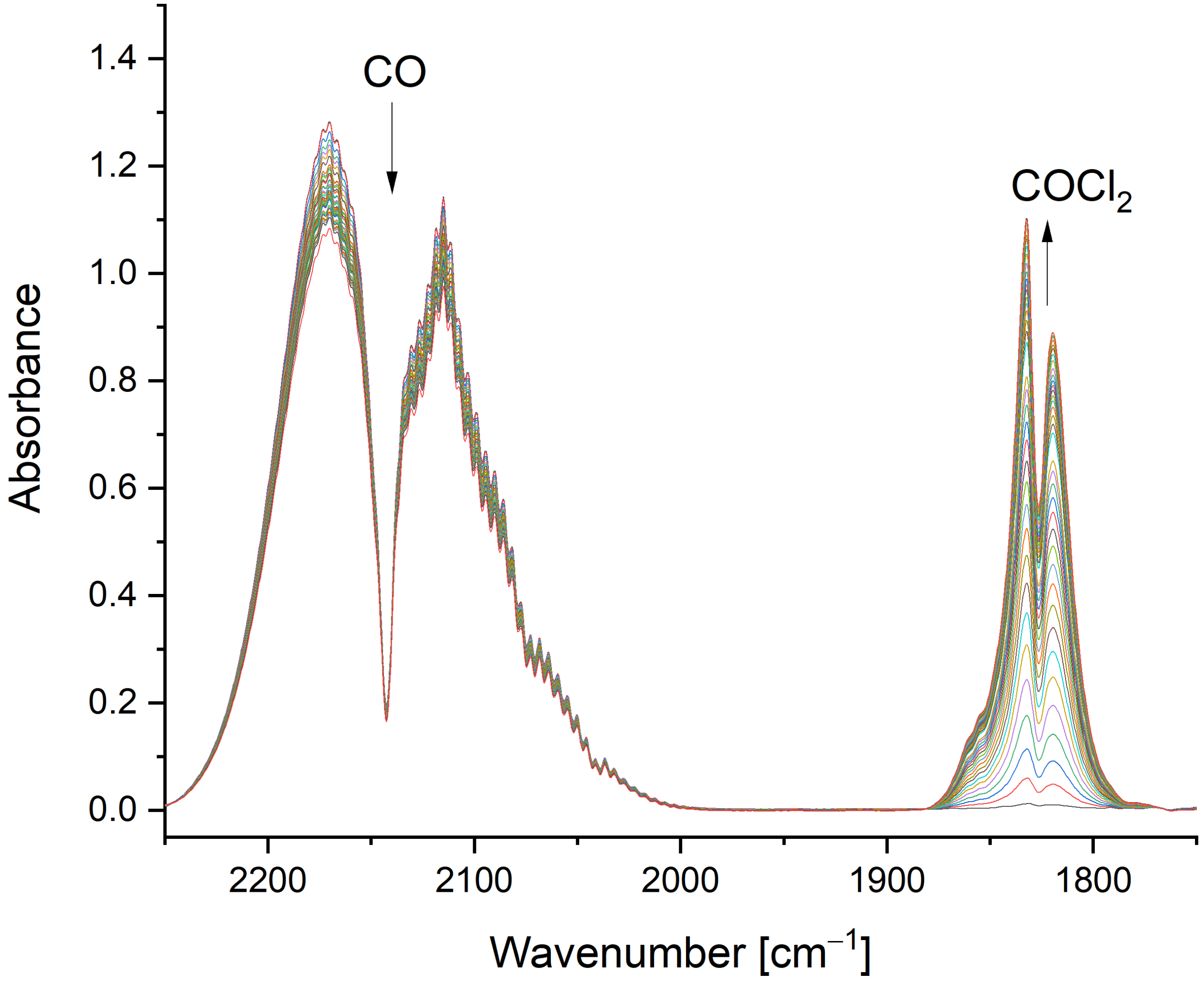

Fig. S12. Time-dependent gas-phase IR spectra of the gaseous constituents obtained from the reaction of $[NEt_3Me][Cl_3]$ + CO. Spectra were recorded after 0, 5, 10, 15, 20, 25, 30, 35, 40, 45, 50, 55, 60, 65, 70, 75, 80, 85 98, 103, 108, 113, 123, 128, 133, 138, 144, 154, 164, 174, 184, 194, 204, 214, 224 min.

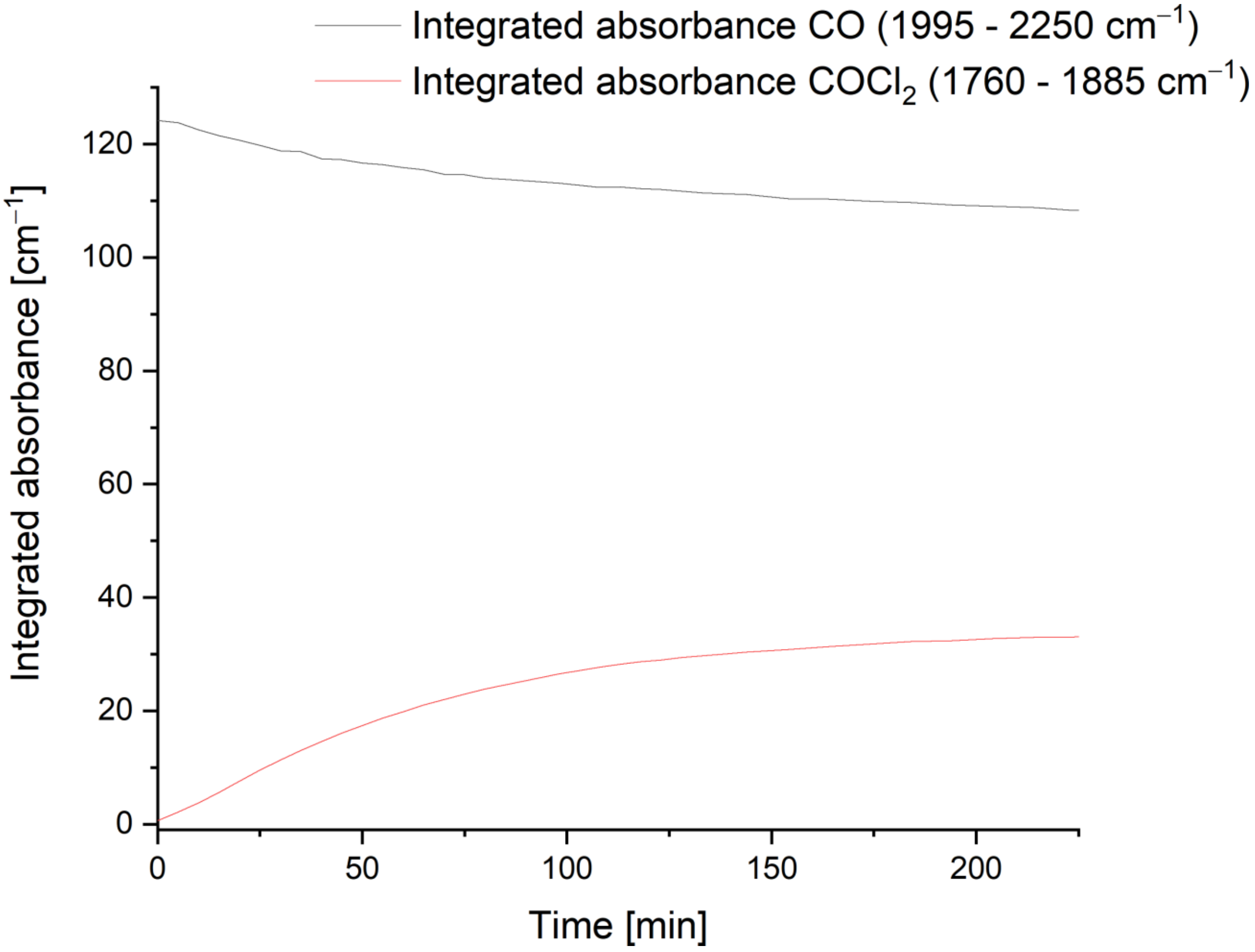

Fig. S13. Time-dependent integrated gas-phase IR absorbances for the CO stretching bands of CO (black line, 1995 – 2250 cm^{-1}) and $COCl_2$ (red line, 1760 – 1885 cm^{-1}) taken for the reaction of $[NEt_3Me][Cl_3]$ + CO at reaction times between 0 and 224 min.

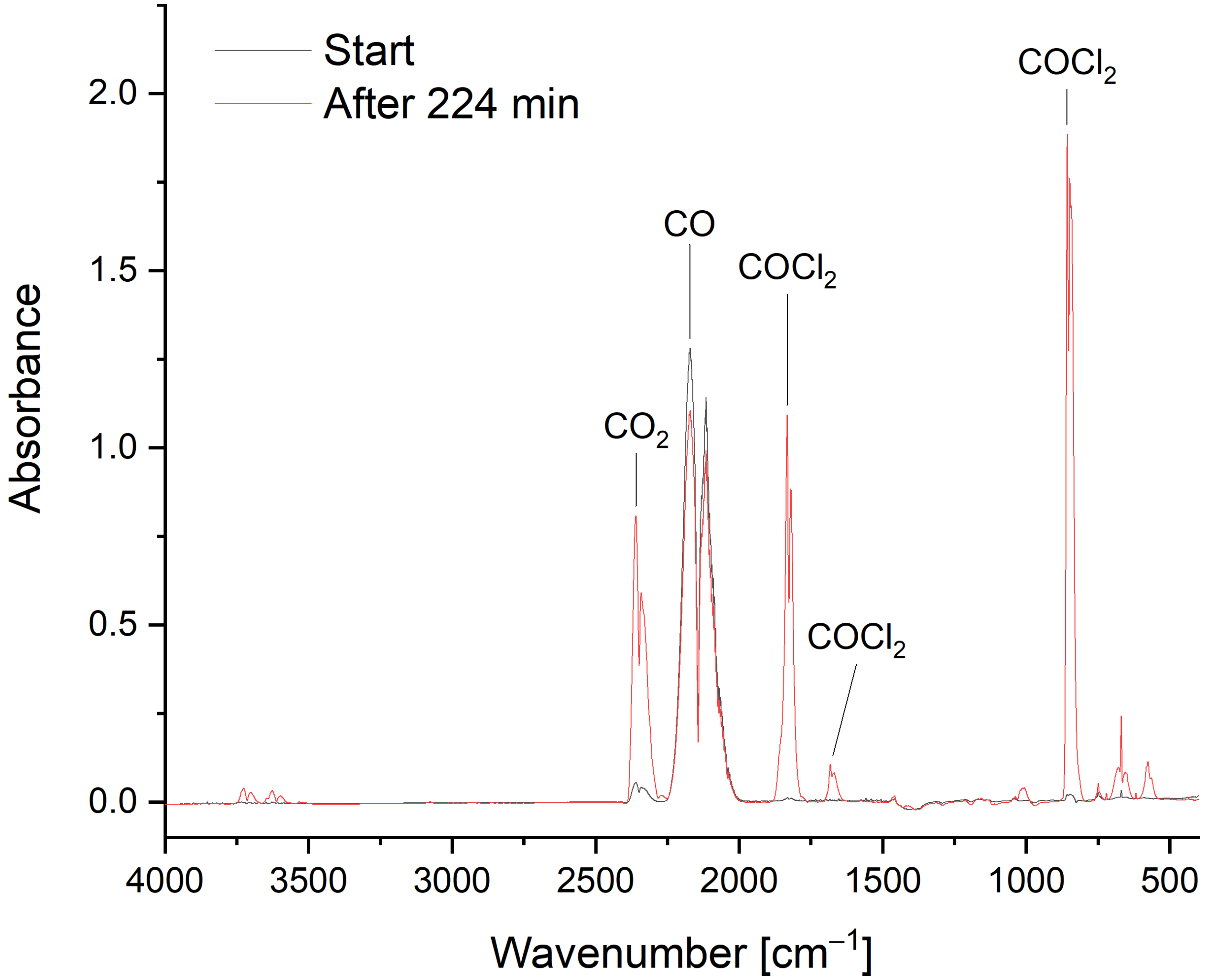

Fig. S14. Gas-phase IR spectra of the gaseous constituents obtained from the reaction of [NEt_3Me][Cl_3] + CO and at reaction times of 0 (black spectrum) and 224 min (red spectrum), respectively.

[NEt$_4$]Cl + CO + Cl$_2$ (flow)

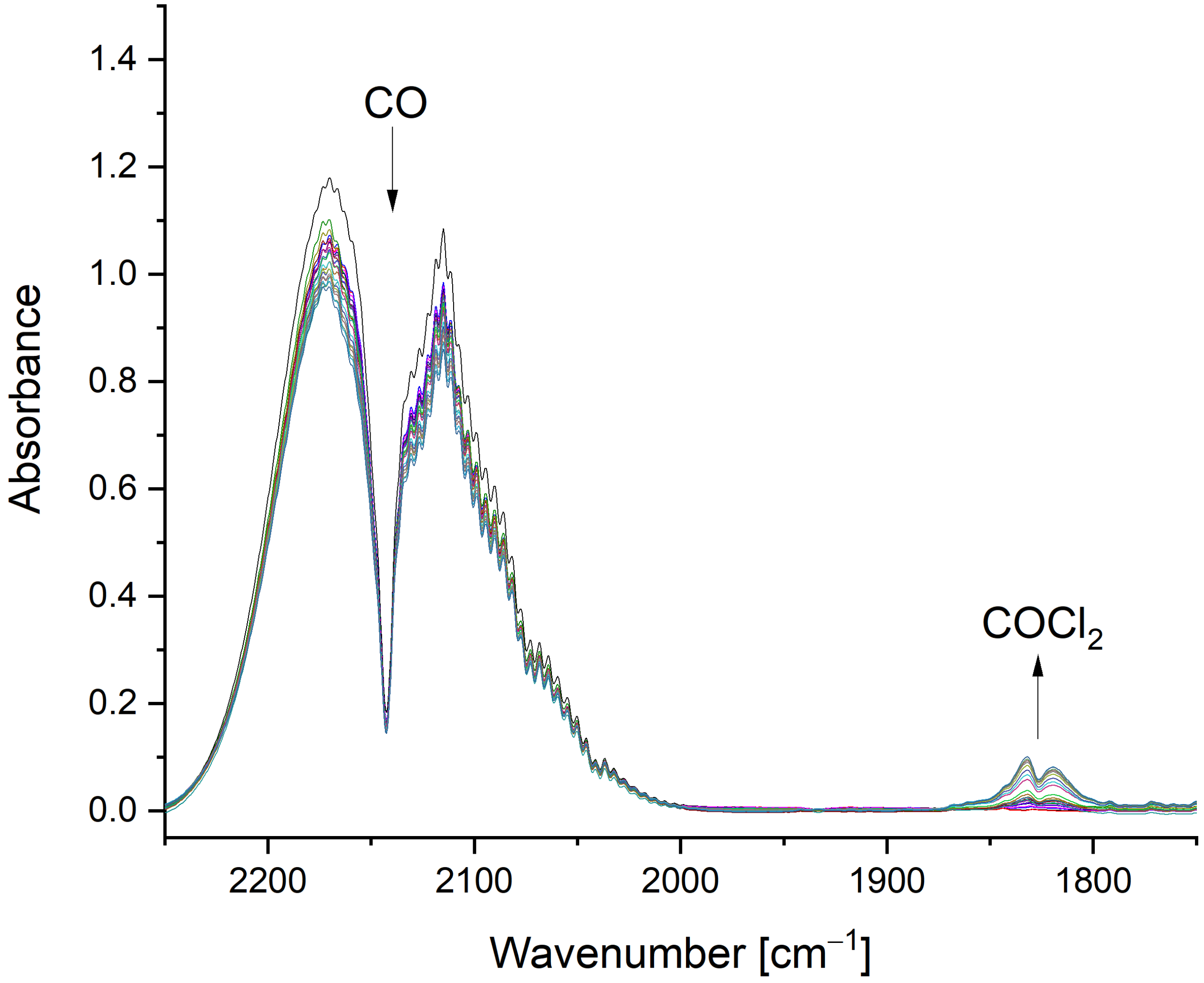

Fig. S15. Time-dependent gas-phase IR spectra of the gaseous constituents obtained from the reaction of [NEt$_4$]Cl + CO + Cl$_2$. Spectra were recorded after 0, 1, 6, 11, 21, 27, 32, 37, 42, 47, 52, 65, 77, 137, 197, 257, 317, 377, 437, 497, 558, 628 min.

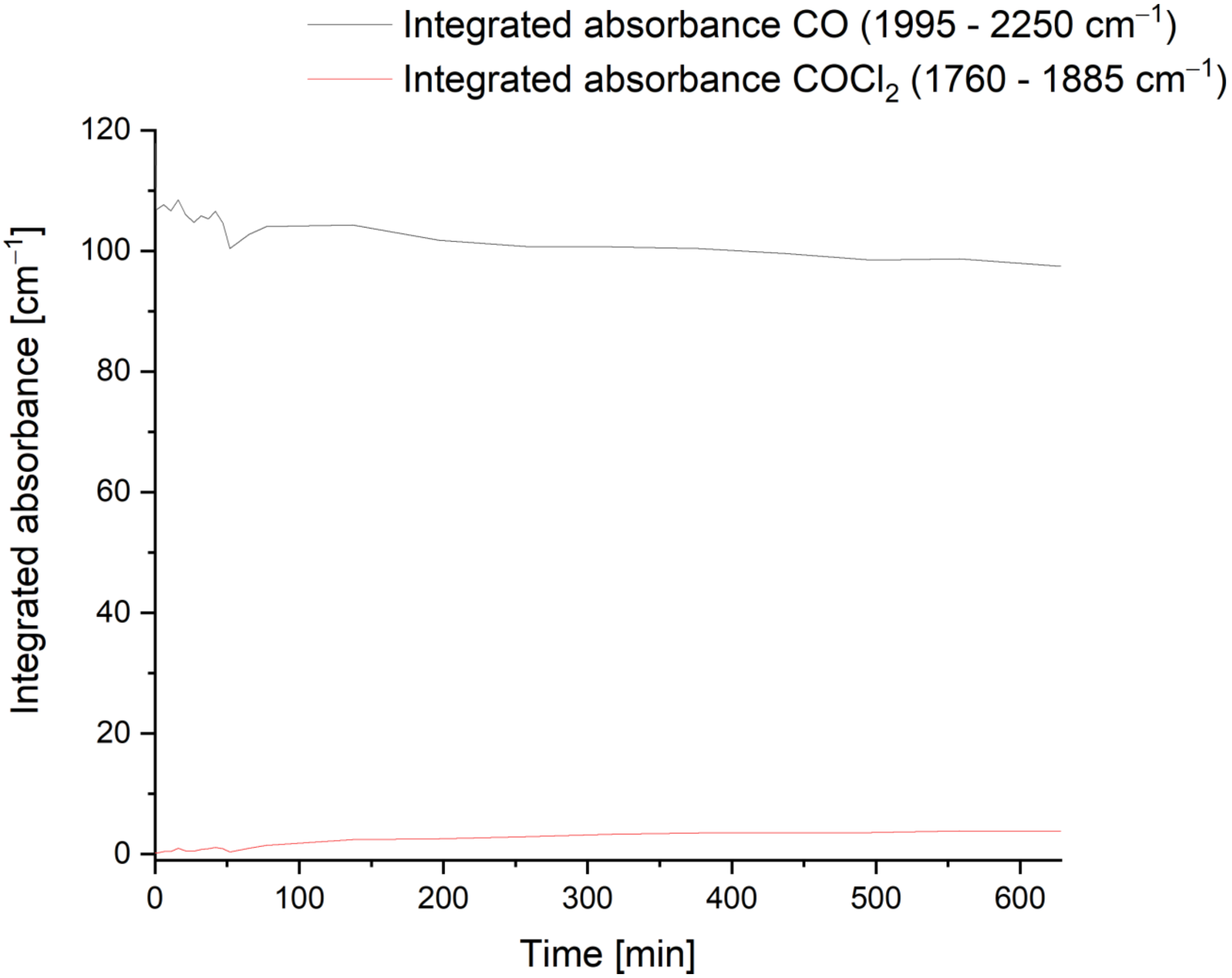

Fig. S16. Time-dependent integrated gas-phase IR absorbances for the CO stretching bands of CO (black line, 1995 – 2250 cm^{-1}) and $COCl_2$ (red line, 1760 – 1885 cm^{-1}) taken for the reaction of $[NEt_4]Cl + CO + Cl_2$ at reaction times 0 and 628.

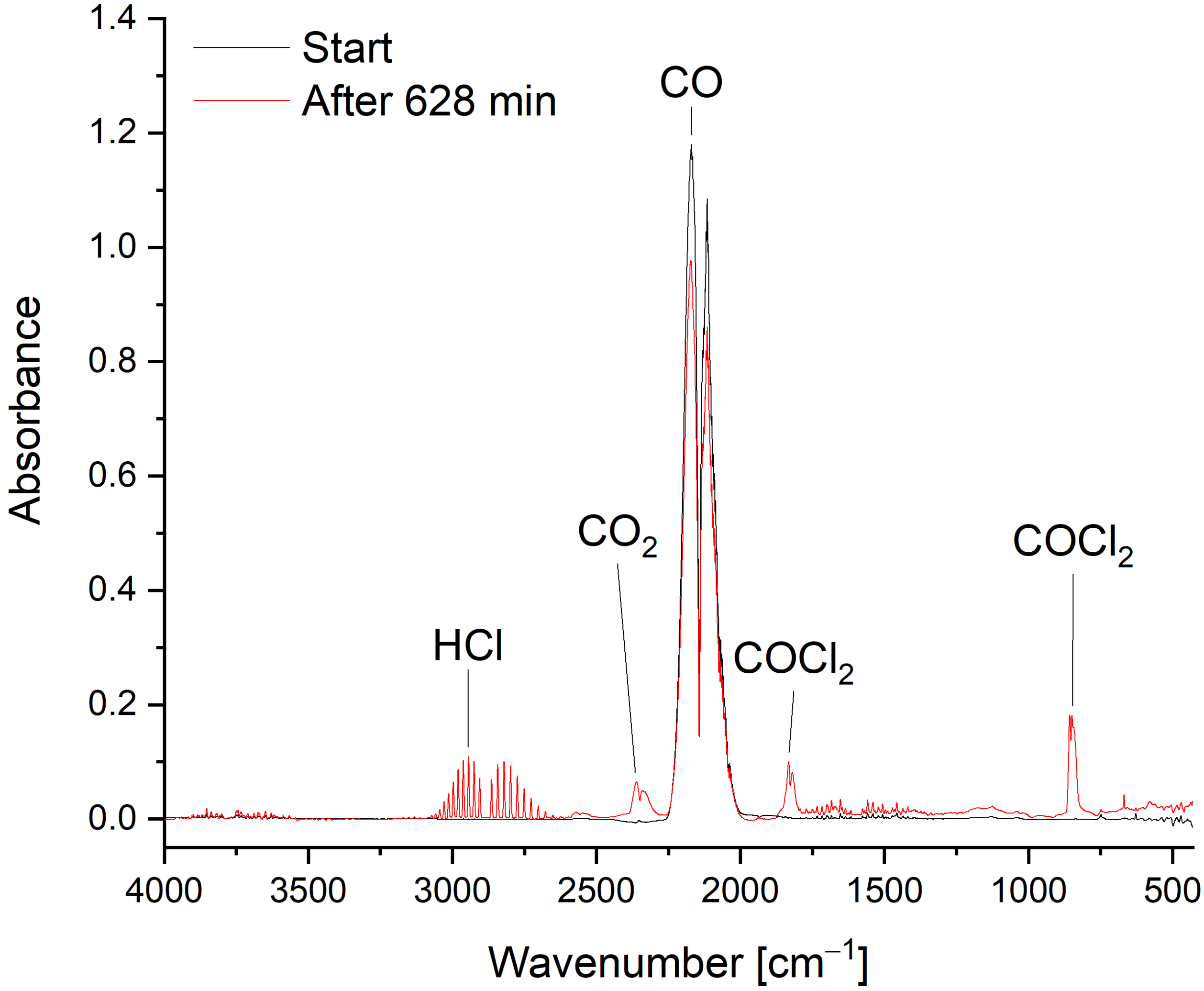

Fig. S17. Gas-phase IR spectra of the gaseous constituents obtained in the reaction of [NEt_4]Cl + CO + Cl_2 at reaction times of 0 and 628, respectively.

CO + Cl_2 (under exclusion of light, flow)

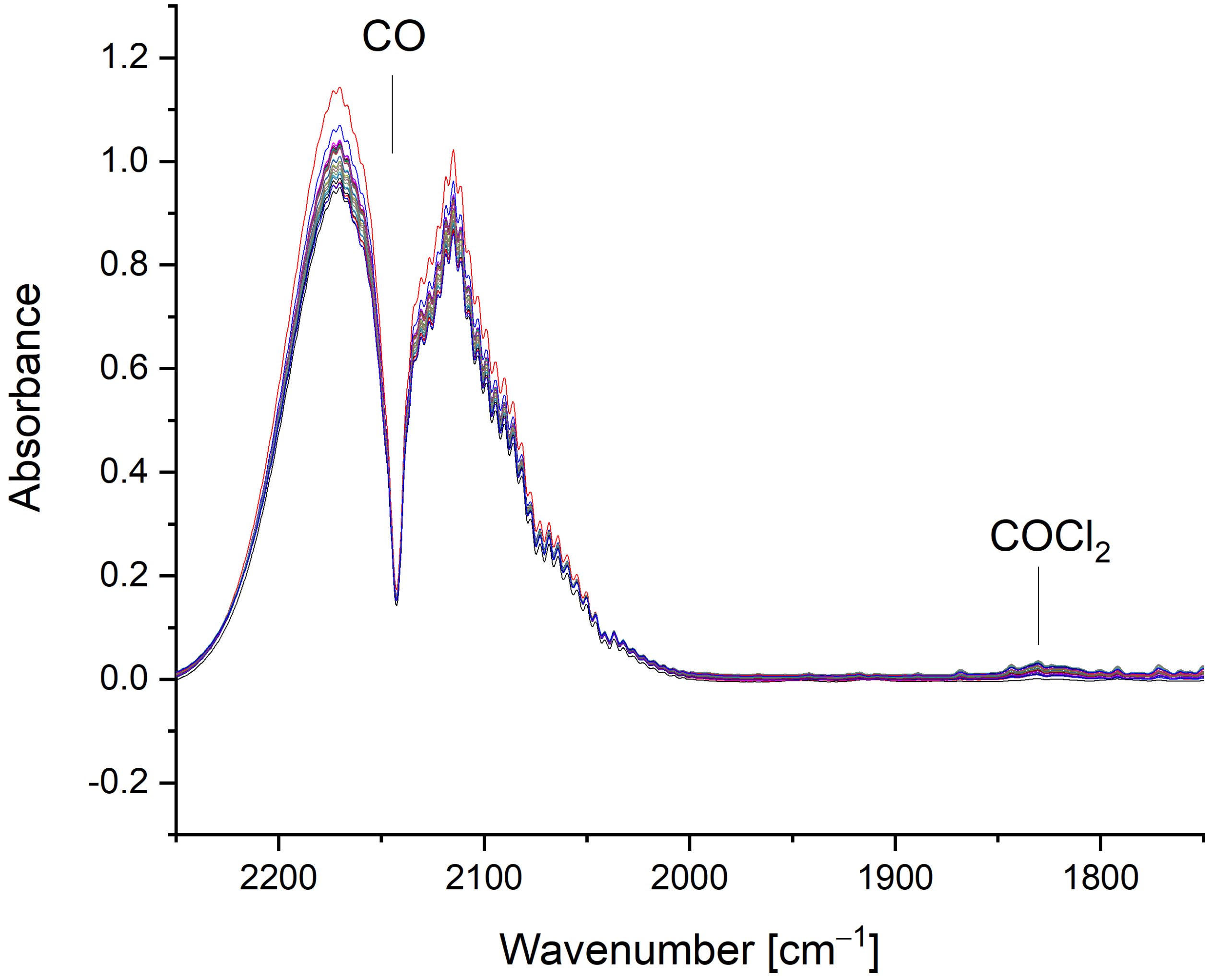

Fig. S18. Time-dependent gas-phase IR spectra of the gaseous constituents obtained from the reaction of Cl_2 + CO. Spectra were recorded after 0, 4, 9, 14, 18, 19, 34, 49, 64, 79, 94, 109, 124, 139, 154, 261, 262, 323, 384, 445, 506, 567, 628, 689, 750, 811 min.

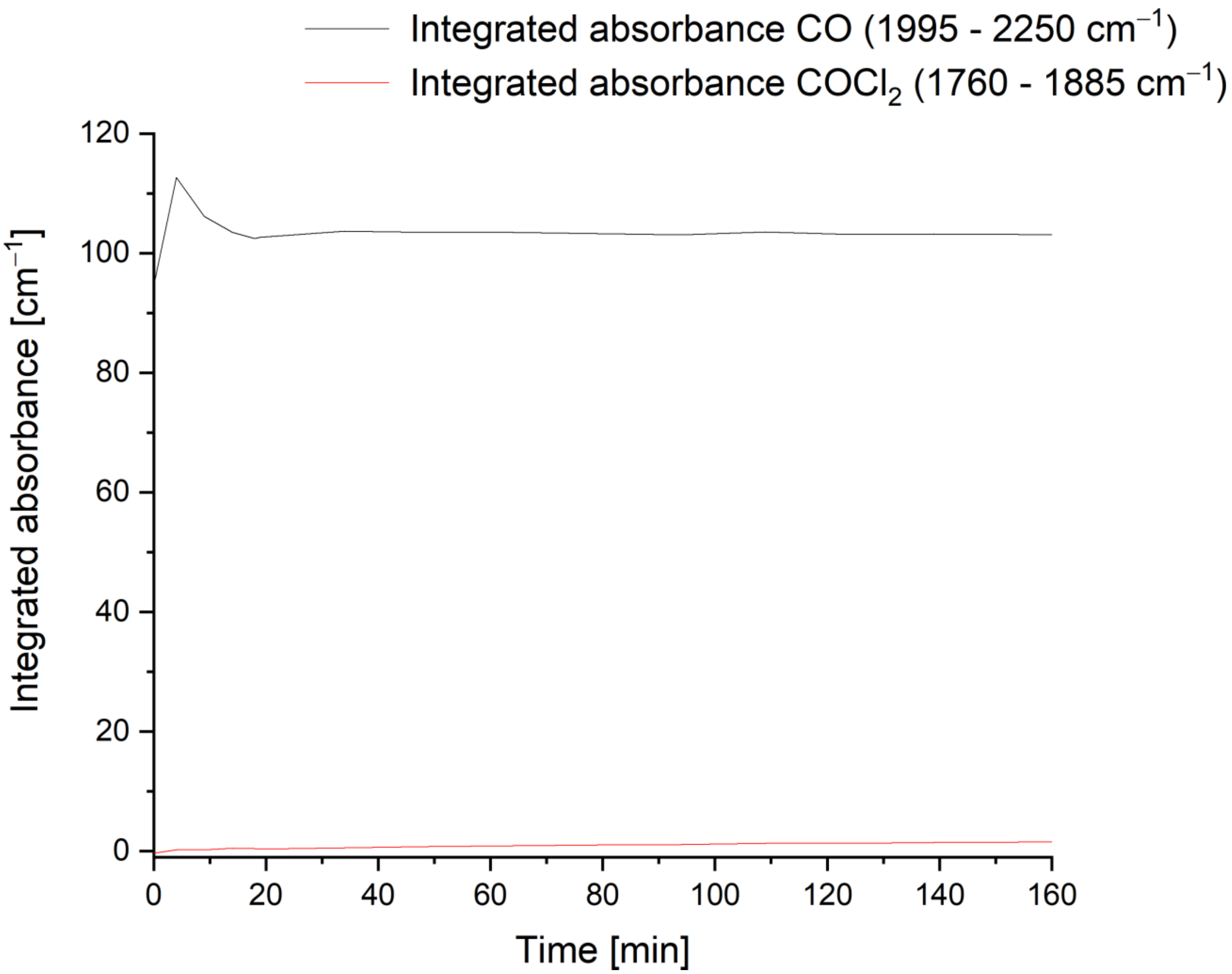

Fig. S19. Time-dependent integrated gas-phase IR absorbances for the CO stretching bands of CO (black line, 1995 – 2250 cm^{-1}) and $COCl_2$ (red line, 1760 – 1885 cm^{-1}) taken for the reaction CO + Cl_2 at reaction times 0 and 628.

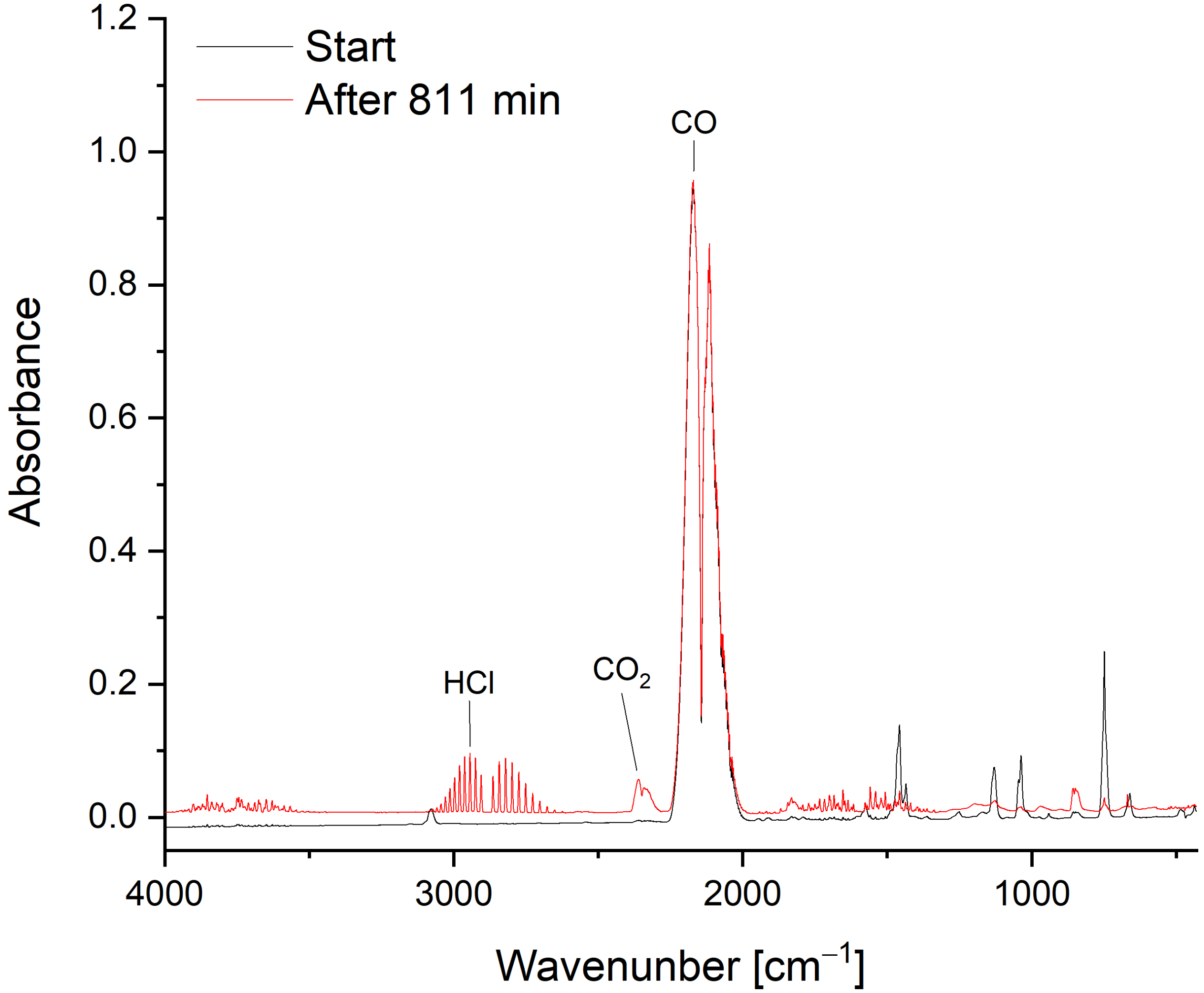

Fig. S20. Gas-phase IR spectra of the gaseous constituents obtained in the reaction of CO + Cl_2 at reaction times of 0 and 811 min, respectively.

CO + Cl_2 (without exclusion of light, flow)

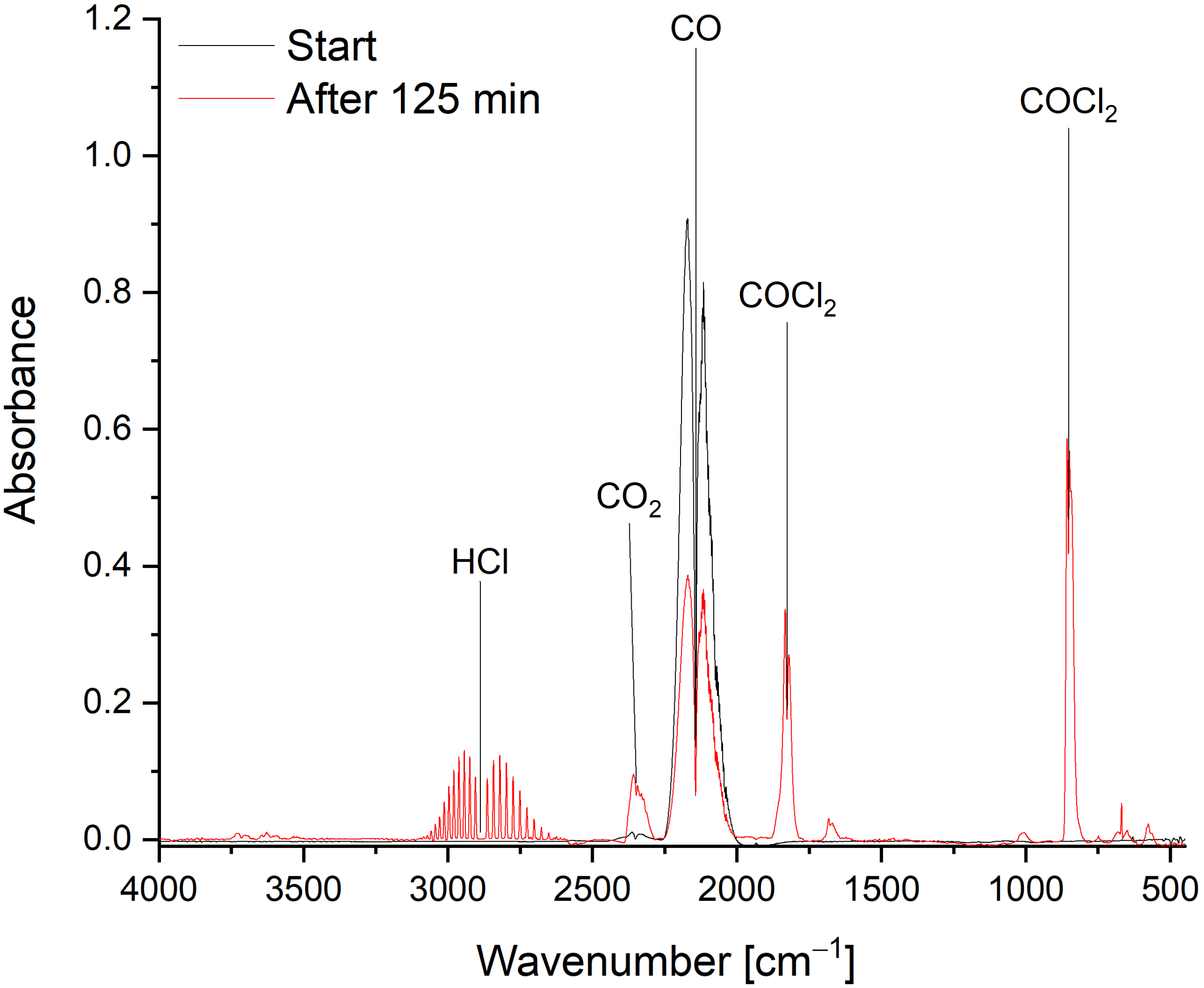

Fig. S21. Gas-phase IR spectra of the gaseous constituents obtained in the reaction of CO + Cl_2 at reaction times of 0 and 125 min, respectively, with continuous UV/Vis measurements.

Comparison of the $COCl_2$ formation using different catalyst systems as well as the uncatalysed reaction

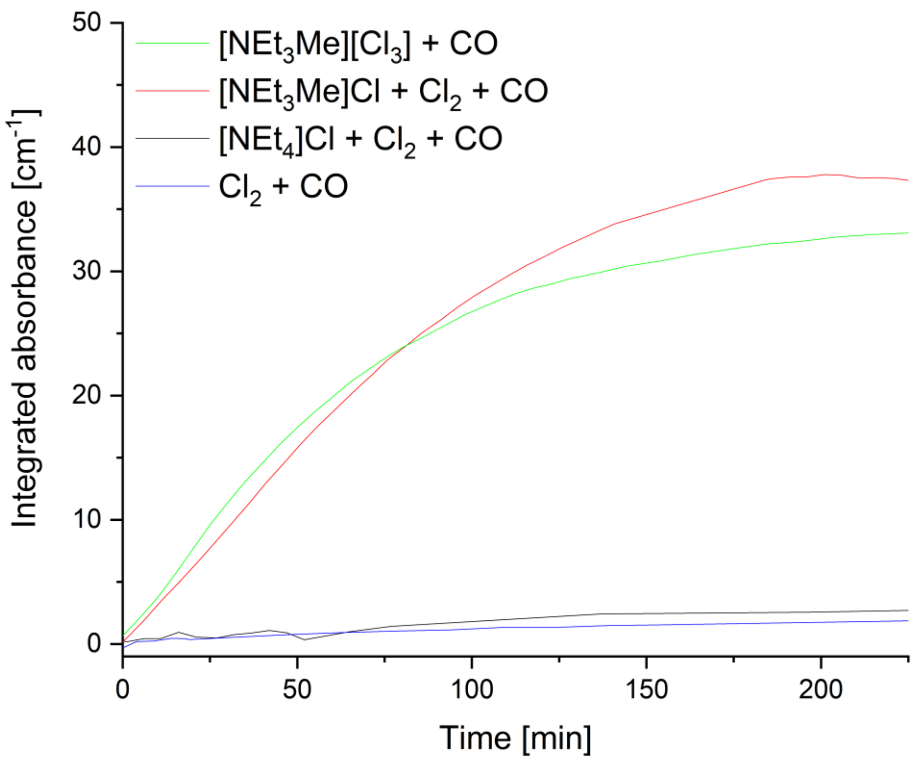

Fig. S22. Comparison of the $COCl_2$ formation using different catalyst systems. The concentration of $COCl_2$ is estimated by integrating the IR absorbance of the carbonyl stretching vibration of $COCl_2$ (1760 – 1885 cm^{-1}). The following conditions were used: dashed - dotted line: $[NEt_3Me][Cl(Cl_2)_{1.50}]$ (0.783 g, 3.033 mmol $[NEt_3Me]Cl$ + 4.555 mmol Cl_2), 20 mL *o*DCB, 1000 mbar CO (22.32 mmol); dashed line: $[NEt_3Me]Cl$ (0.460 g, 3.033 mmol), 20 mL *o*DCB, 1000 mbar CO (22.32 mmol), and 208 mbar Cl_2 (4.65 mmol); dotted line: $[NEt_4]Cl$ (0.230 g, 1.393 mmol), 850 mbar CO (20.20 mmol), 150 mbar Cl_2 (3.530 mmol); continuous line: 850 mbar CO (20.20 mmol, 150 mbar Cl_2 (3.53 mmol) in 20 mL *o*DCB (under exclusion of light). See Materials and Methods for detailed description.

[NEt3Me]Cl + Cl2 + CH4

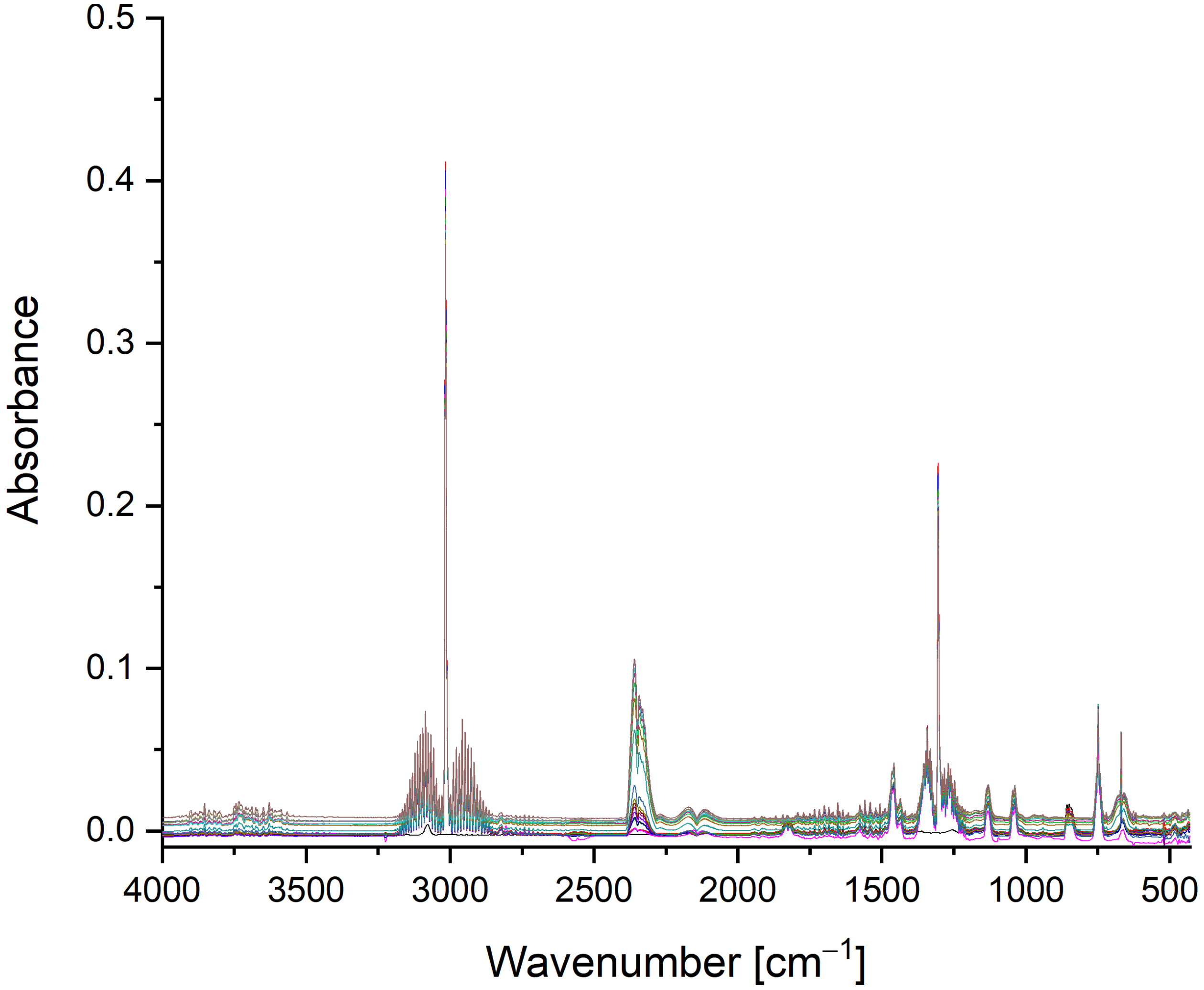

Fig. S23. Gas-phase IR spectra of the gaseous constituents obtained during the reaction of $[NEt_3Me]Cl + Cl_2 + CH_4$. Spectra were recorded after 0, 3, 6, 9, 12, 15, 19, 22, 25, 28, 39, 40, 100, 161, 221, 282, 342, 403, 363, 524 min.

$PhNH_2$ + $COCl_2$ → PhNCO

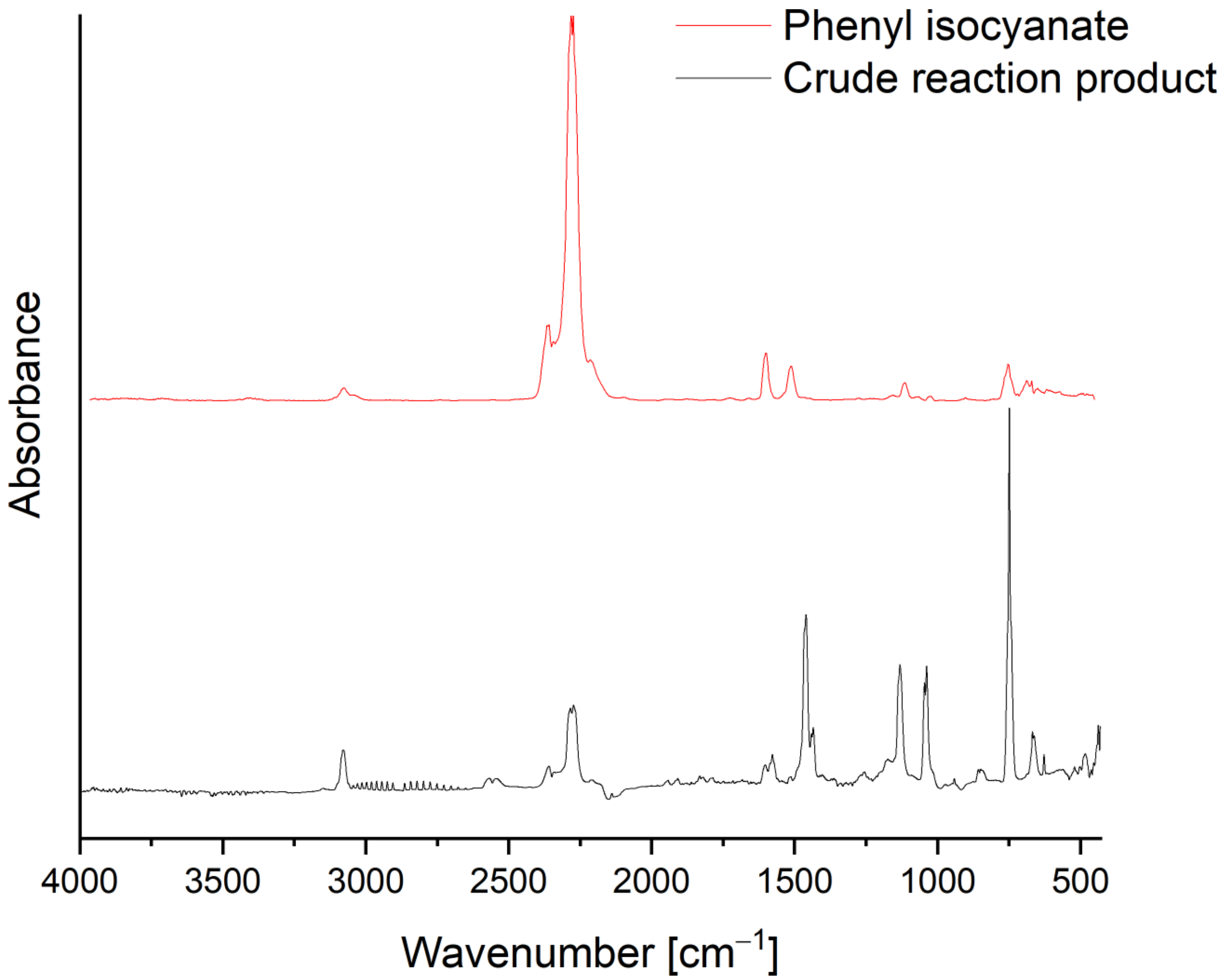

Fig. S24. Comparison of the gas-phase IR spectrum of the crude reaction product obtained from the reaction $PhNH_2$ + $COCl_2$ (black) with the gas-phase IR spectrum of neat phenyl isocyante (red). (*56*)

Vapor Pressure Curve of pristine [NEt_3Me][$Cl(Cl_2)_x$]

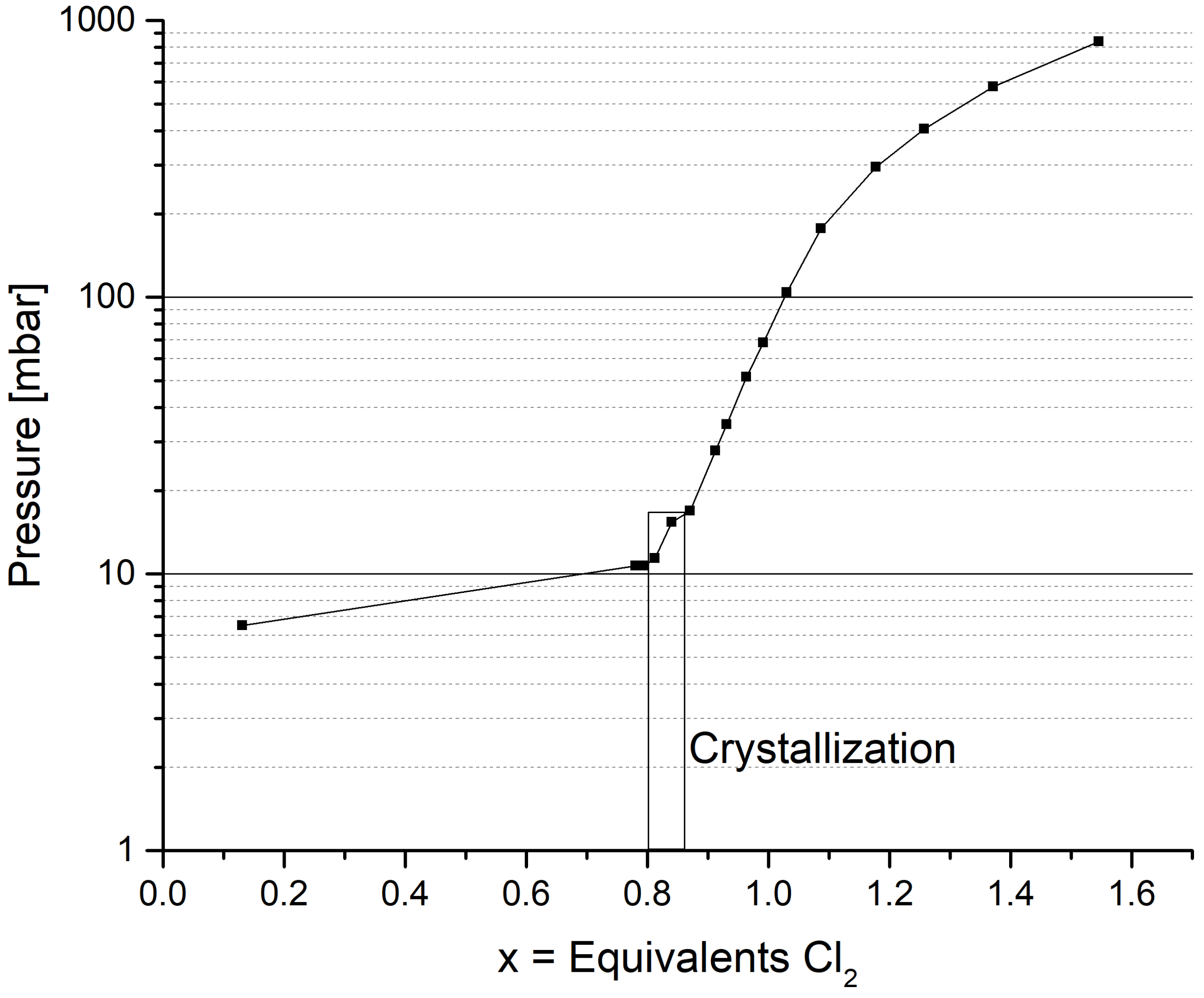

Fig. S25. Vapor pressure of pristine [NEt_3Me][$Cl(Cl_2)_x$] depending on x.

Half-life time estimation for the reaction of [NEt_3Me]Cl_3 and CO in oDCB

Description of the procedure

The half-life time for the CO consumption of the reaction of [NEt_3Me]Cl_3 with CO in oDCB, was estimated in three different experiments, carried out under similar conditions and amount of starting materials (see table S1), but different reaction times. The reaction was carried out as followed:

[NEt_3Me]Cl was loaded into a 250 mL Schlenk flask, dried *in vacuo* at 150 °C for 1 hour and suspended in 10 mL dry *o*-Dichlorobenzene (*o*DCB). The solution was degassed and chlorine was added until the system retained a pressure of 200 mbar. 800 mbar of CO were added to the flask and the reaction mixture was stirred, under the exclusion of light, for 62, 202 and 1000 min, respectively. CO was then removed from the reaction mixture by freeze-pump-thaw-degassing (CO is the only reactant with a residual vapor pressure at a temperature of –196 °C). To determine the amount of consumed CO the flask was weighted bevor and after the degassing.

Table S4. Amount of chemicals used in the reactions.

**Batch**	**1**	**2**	**3**
***m*([NEt_3Me]Cl) [mg]**	369	363	360
***n*([NEt_3Me]Cl) [mmol]**	2.43	2.39	2.37
***m*(Cl_2) [mg]**	551	560	567
***n*(Cl_2) [mmol]**	7.77	7.90	8.00
***m*(CO) [mg]**	241	248	247
***n*(CO) [mg]**	8.60	8.85	8.82
***m*(CO (used)) [mg]**	27	94	190
***m*(CO (left)) [mg]**	214	154	57
***n*(CO (left)) [mmol]**	7.64	5.50	2.04
**reaction time [min]**	62	202	1000

The uncertainties of measured masses and reaction times are estimated to be ±5 mg (due to residual grease from the joints) and ±5 min, respectively. The following error propagation is assumed:

$$\delta m = \delta n = \delta\left(\frac{1}{n}\right) \tag{1}$$

For plotting 1/ amount of substance vs. time a linear correlation was obtained indicating a second order reaction. Therefore the expression for the time dependence of the concentration for a second order reaction was used (assuming that $[CO]_0=[Cl_2]_0$):

$$\frac{1}{[\mathrm{CO}]} = \frac{1}{[\mathrm{CO}]_0} + kt \qquad (2)$$

The half-life of a second order reaction is given by:

$$t_{1/2} = \frac{1}{[\mathrm{CO}]_0 \cdot k} \qquad (3)$$

and using a linear fit (see Fig. S25) k was determined to be (0.38 ± 0.01) mol^{-1} min^{-1} and $t_{1/2}$ was determined to be (287 ± 14) min.

Plots

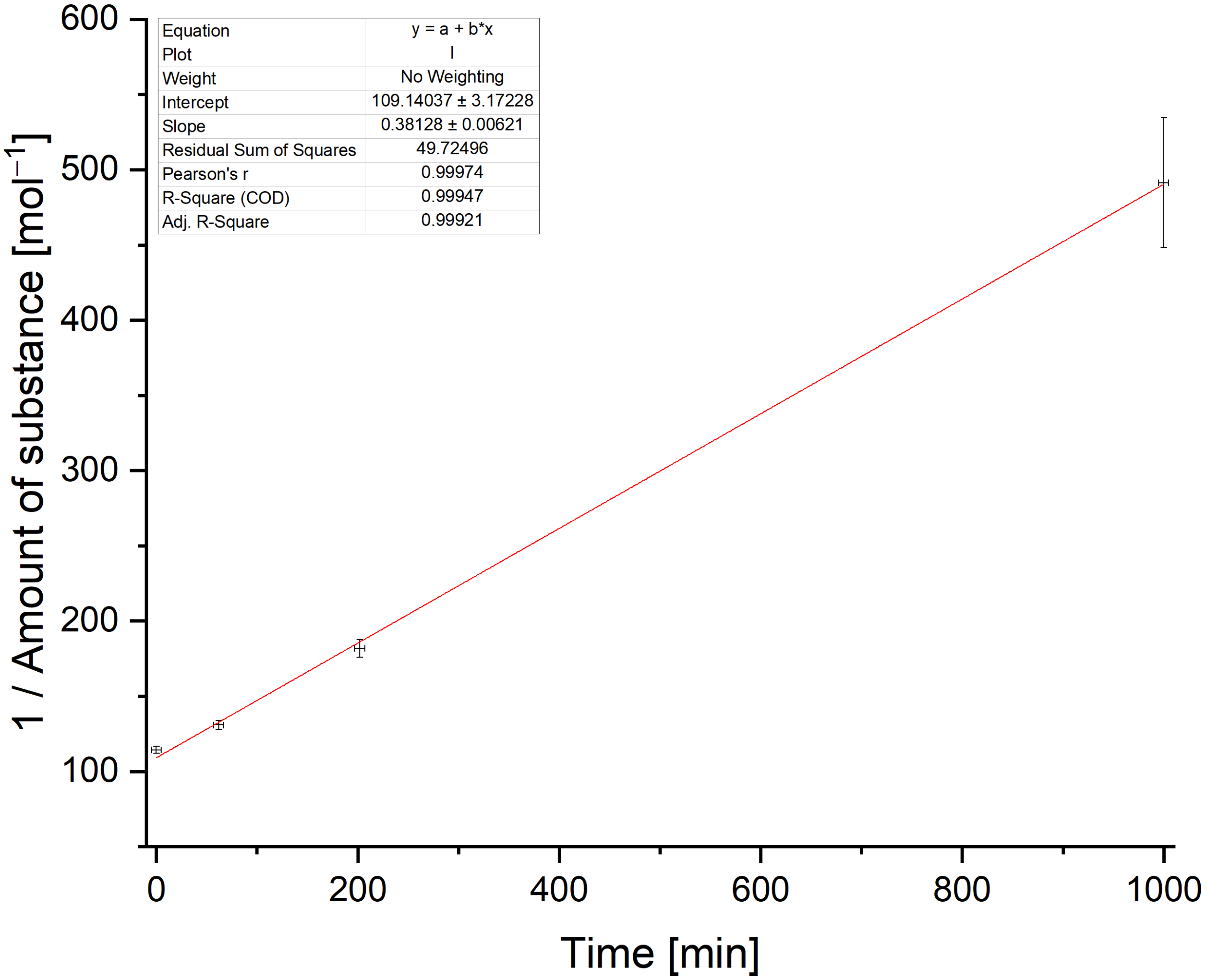

Fig. S26. Plot of 1 / *n*(CO) vs time.

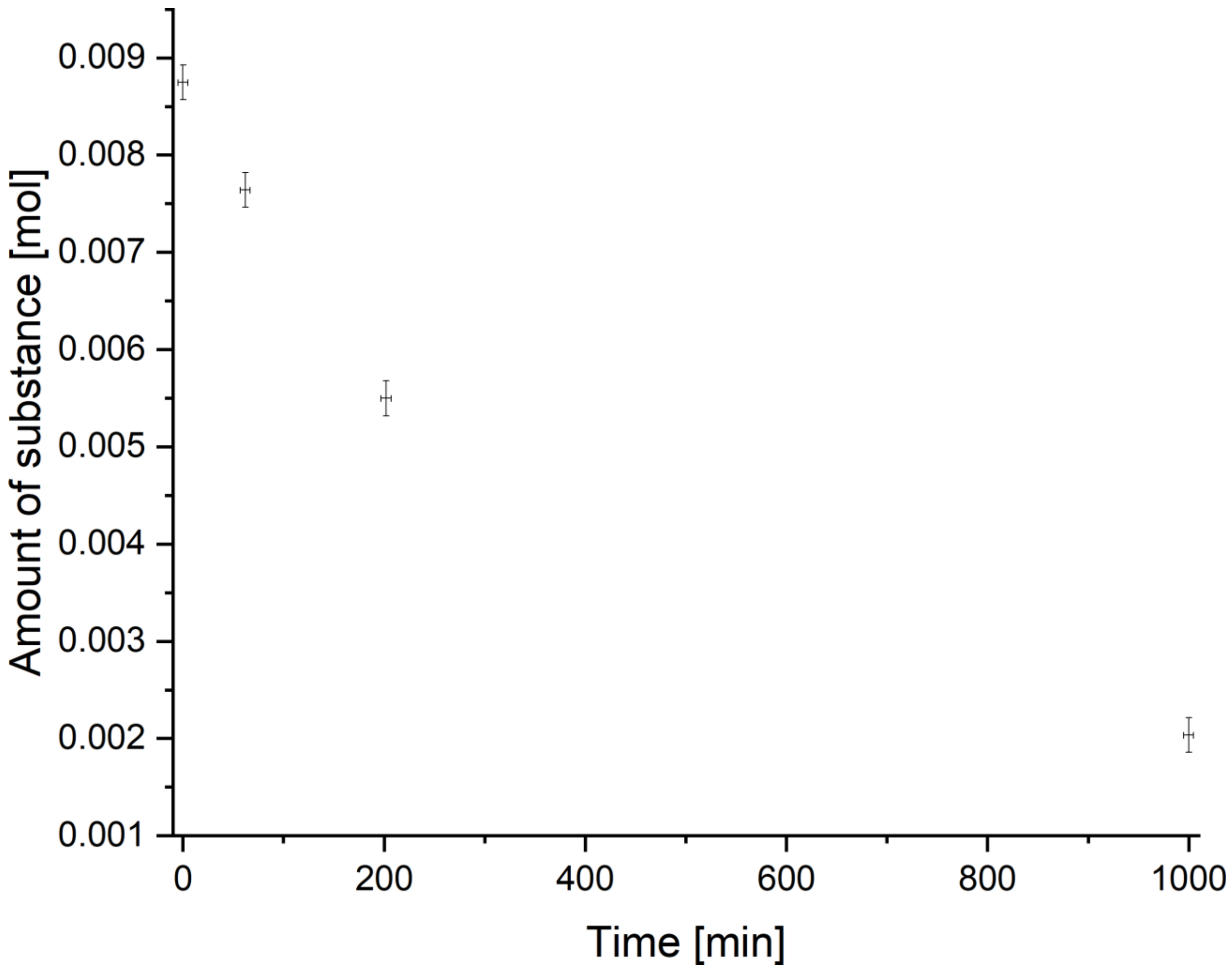

Fig. S27. Plot of *n*(CO) vs time

Interpretation of the obtained values

Some rather harsh assumption had to be made to obtain the values described above. The reaction between $[NEt_3Me][Cl_3]$ and CO takes place in a rather complicated reaction media consisting of three phase (gas phase (Cl_2 and CO), *o*DCB solution (solved Cl_2, maybe small amounts of $[Cl_3]^-$ and CO) and the $[NEt_3Me][Cl_3]$ ionic liquid phase). It is not completely clear in which phase the reaction takes place but it is most likely that small amounts of CO dissolve in $[NEt_3Me][Cl_3]$ and react there. In this system the $[Cl_3]^-$ will be in large excess over the CO which makes the assumption that n (CO) ≈ n (Cl_2) rather unprecise. If the mixing of the reactants is fast the concentration of CO in $[NEt_3Me][Cl_3]$ should be nearly constant and should only slowly decrease when the partial pressure of CO in the gas phase decreases. Additionally, the concentration of $[Cl_3]^-$ will also stay rather constant since consumed Cl_2 will be replaced by Cl_2 from dissolved Cl_2 from the oDCB phase or from the gas phase. Due to this many equilibria between the phases a rather complex kinetic can be expected. Nevertheless, the plot of 1/ n(CO) vs t shows a rather good linear behavior and at least the determined $t_{1/2}$ of (287 ± 14) min (for this defined system) can be used as a good estimate for the speed of the reaction. A half-life of ca. 5 h also correlates well to the observed reaction time of ca. 2 days for a full conversion of the Cl_2 to $COCl_2$.

Movie S1. Synthesis and handling of $[NEt_3Me][Cl(Cl_2)_{1.45}]$

REFERENCES AND NOTES

1. J. Davy, VI. On a gaseous compound of carbonic oxide and chlorine. *Phil. Trans. R. Soc.* **102**, 144–151 (1812).

2. L. Cotarca, C. Lange, K. Meurer, J. Pauluhn, *Ullmann's Encyclopedia of Industrial Chemistry* (Phosgene, 2019), pp. 1–30.

3. G. E. Rossi, J. M. Winfield, N. Meyer, D. H. Jones, R. H. Carr, D. Lennon, Phosgene formation via carbon monoxide and dichlorine reaction over an activated carbon catalyst: Towards a reaction model. *Appl. Catal. Gen.* **609**, 117900 (2021).

4. N. K. Gupta, A. Pashigreva, E. A. Pidko, E. J. M. Hensen, L. Mleczko, S. Roggan, E. E. Ember, J. A. Lercher, Bent carbon surface moieties as active sites on carbon catalysts for phosgene synthesis. *Angew. Chem. Int. Ed.* **55**, 1728–1732 (2016).

5. N. K. Gupta, B. Peng, G. L. Haller, E. E. Ember, J. A. Lercher, Nitrogen modified carbon nano-materials as stable catalysts for phosgene synthesis. *ACS Catal.* **6**, 5843–5855 (2016).

6. G. E. Rossi, J. M. Winfield, C. J. Mitchell, N. Meyer, D. H. Jones, R. H. Carr, D. Lennon, Phosgene formation via carbon monoxide and dichlorine reaction over an activated carbon catalyst: Reaction kinetics and mass balance relationships. *Appl. Catal. Gen.* **602**, 117688 (2020).

7. C. J. Mitchell, W. van der Borden, K. van der Velde, M. Smit, R. Scheringa, K. Ahrika, D. H. Jones, Selection of carbon catalysts for the industrial manufacture of phosgene. *Cat. Sci. Technol.* **2**, 2109 (2012).

8. S. K. Ajmera, M. W. Losey, K. F. Jensen, M. A. Schmidt, Microfabricated packed-bed reactor for phosgene synthesis. *AIChE J.* **47**, 1639–1647 (2001).

9. L. Khachatryan, B. Dellinger, Formation of chlorinated hydrocarbons from the reaction of chlorine atoms and activated carbon. *Chemosphere* **52**, 709–716 (2003).

10. K. Sonnenberg, L. Mann, F. A. Redeker, B. Schmidt, S. Riedel, Polyhalogen and polyinterhalogen anions from fluorine to iodine. *Angew. Chem. Int. Ed.* **59**, 5464–5493 (2020).

11. H. Keil, D. Sonnenberg, C. Müller, R. Herbst-Irmer, H. Beckers, S. Riedel, D. Stalke, Insights in the topology and the formation of a genuine ppσ bond: Experimental and computed electron densities in mono anionic trichlorine $[Cl_3]^-$. *Angew. Chem.* **60**, 2569–2573 (2020).

12. M. Paven, Y. Schiesser, R. Weber, G. Langstein, V. Trieu, S. Hasenstab-Riedel, N. Schwarze, S. Steinhauer, Storage medium and a method of separating, storage and transportation of chlorine derived from chlorine-containing gases, WO2019215037A1 (2018).

13. S. Grimme, Improved second-order Møller–Plesset perturbation theory by separate scaling of parallel- and antiparallel-spin pair correlation energies. *J. Chem. Phys.* **118**, 9095–9102 (2003).

14. S. Dapprich, I. Komáromi, K.S. Byun, K. Morokuma, M. J. Frisch, A new ONIOM implementation in Gaussian98. Part I. The calculation of energies, gradients, vibrational frequencies and electric field derivatives. *J. Mol. Struct.* **461–462**, 1–21 (1999).

15. M. J. Frisch, G. W. Trucks, H. B. Schlegel, G. E. Scuseria, M. A. Robb, J. R. Cheeseman, G. Scalmani, V. Barone, G. A. Petersson, H. Nakatsuji, X. Li, M. Caricato, A. V. Marenich, J. Bloino, B. G. Janesko, R. Gomperts, B. Mennucci, H. P. Hratchian, J. V. Ortiz, A. F. Izmaylov, J. L. Sonnenberg, D. Williams-Young, F. Ding, F. Lipparini, F. Egidi, J. Goings, B. Peng, A. Petrone, T. Henderson, D. Ranasinghe, V. G. Zakrzewski, J. Gao, N. Rega, G. Zheng, W. Liang, M. Hada, M. Ehara, K. Toyota, R. Fukuda, J. Hasegawa, M. Ishida, T. Nakajima, Y. Honda, O. Kitao, H. Nakai, T. Vreven, K. Throssell, J. A. Montgomery, Jr., J. E. Peralta, F. Ogliaro, M. J. Bearpark, J. J. Heyd, E. N. Brothers, K. N. Kudin, V. N. Staroverov, T. A. Keith, R. Kobayashi, J. Normand, K. Raghavachari, A. P. Rendell, J. C. Burant, S. S. Iyengar, J. Tomasi, M. Cossi, J. M. Millam, M. Klene, C. Adamo, R. Cammi, J. W. Ochterski, R. L. Martin, K. Morokuma, O. Farkas, J. B. Foresman, and D. J. Fox, *Gaussian 16* (Gaussian Inc., 2016).

16. H.-J. Werner, P. J. Knowles, G. Knizia, F. R. Manby, M. Schütz, Molpro: A general-purpose quantum chemistry program package. *WIREs Comput. Mol. Sci.* **2**, 242–253 (2012).

17. H.-J. Werner, P. J. Knowles, G. Knizia, F. R. Manby, M. Schütz, P. Celani, W. Györffy, D. Kats, T. Korona, R. Lindh, A. Mitrushenkov, G. Rauhut, K. R. Shamasundar, T. B. Adler, R. D. Amos, S. J. Bennie, A. Bernhardsson, A. Berning, D. L. Cooper, M. J. O. Deegan, A. J. Dobbyn, F. Eckert, E.

Goll, C. Hampel, A. Hesselmann, G. Hetzer, T. Hrenar, G. Jansen, C. Köppl, S. J. R. Lee, Y. Liu, A. W. Lloyd, Q. Ma, R. A. Mata, A. J. May, S. J. McNicholas, W. Meyer, T. F. Miller III, M. E. Mura, A. Nicklass, D. P. O'Neill, P. Palmieri, D. Peng, K. Pflüger, R. Pitzer, M. Reiher, T. Shiozaki, H. Stoll, A. J. Stone, R. Tarroni, T. Thorsteinsson, M. Wang, M. Welborn, *MOLPRO, version 2019.2, a package of ab initio programs*.

18. T. H. Dunning Jr, Gaussian basis sets for use in correlated molecular calculations. I. The atoms boron through neon and hydrogen. *J. Chem. Phys.* **90**, 1007–1023 (1989).

19. R. A. Kendall, T. H. Dunning, R. J. Harrison, Electron affinities of the first-row atoms revisited. Systematic basis sets and wave functions. *J. Chem. Phys.* **96**, 6796–6806 (1992).

20. D. E. Woon, T. H. Dunning Jr, Gaussian basis sets for use in correlated molecular calculations. III. The atoms aluminum through argon. *J. Chem. Phys.* **98**, 1358–1371 (1993).

21. F. Weigend, A fully direct RI-HF algorithm: Implementation, optimised auxiliary basis sets, demonstration of accuracy and efficiency. *Phys. Chem. Chem. Phys.* **4**, 4285–4291 (2002).

22. S. Miertuš, E. Scrocco, J. Tomasi, Electrostatic interaction of a solute with a continuum. A direct utilizaion of AB initio molecular potentials for the prevision of solvent effects. *Chem. Phys.* **55**, 117–129 (1981).

23. S. Miertus̃, J. Tomasi, Approximate evaluations of the electrostatic free energy and internal energy changes in solution processes. *Chem. Phys.* **65**, 239–245 (1982).

24. J. L. Pascual-ahuir, E. Silla, I. Tuñon, GEPOL: An improved description of molecular surfaces. III. A new algorithm for the computation of a solvent-excluding surface. *J. Comput. Chem.* **15**, 1127–1138 (1994).

25. M. Cossi, V. Barone, R. Cammi, J. Tomasi, Ab initio study of solvated molecules: A new implementation of the polarizable continuum model. *Chem. Phys. Lett.* **255**, 327–335 (1996).

26. E. Cancès, B. Mennucci, J. Tomasi, A new integral equation formalism for the polarizable continuum model: Theoretical background and applications to isotropic and anisotropic dielectrics. *J. Chem. Phys.* **107**, 3032–3041 (1997).

27. V. Barone, M. Cossi, J. Tomasi, A new definition of cavities for the computation of solvation free energies by the polarizable continuum model. *J. Chem. Phys.* **107**, 3210–3221 (1997).

28. B. Mennucci, J. Tomasi, Continuum solvation models: A new approach to the problem of solute's charge distribution and cavity boundaries. *J. Chem. Phys.* **106**, 5151–5158 (1997).

29. B. Mennucci, E. Cancès, J. Tomasi, Evaluation of solvent effects in isotropic and anisotropic dielectrics and in ionic solutions with a unified integral equation method: Theoretical bases, computational implementation, and numerical applications. *J. Phys. Chem. B* **101**, 10506–10517 (1997).

30. V. Barone, M. Cossi, Quantum calculation of molecular energies and energy gradients in solution by a conductor solvent model. *J. Phys. Chem. A* **102**, 1995–2001 (1998).

31. M. Cossi, V. Barone, B. Mennucci, J. Tomasi, Ab initio study of ionic solutions by a polarizable continuum dielectric model. *Chem. Phys. Lett.* **286**, 253–260 (1998).

32. V. Barone, M. Cossi, J. Tomasi, Geometry optimization of molecular structures in solution by the polarizable continuum model. *J. Comput. Chem.* **19**, 404–417 (1998).

33. R. Cammi, B. Mennucci, J. Tomasi, Second-order Møller−Plesset analytical derivatives for the polarizable continuum model using the relaxed density approach. *J. Phys. Chem. A* **103**, 9100–9108 (1999).

34. J. Tomasi, B. Mennucci, E. Cancès, The IEF version of the PCM solvation method: An overview of a new method addressed to study molecular solutes at the QM ab initio level. *J. Mol. Struct. (THEOCHEM)* **464**, 211–226 (1999).

35. M. Cossi, N. Rega, G. Scalmani, V. Barone, Polarizable dielectric model of solvation with inclusion of charge penetration effects. *J. Chem. Phys.* **114**, 5691–5701 (2001).

36. M. Cossi, G. Scalmani, N. Rega, V. Barone, New developments in the polarizable continuum model for quantum mechanical and classical calculations on molecules in solution. *J. Chem. Phys.* **117**, 43–54 (2002).

37. M. Cossi, N. Rega, G. Scalmani, V. Barone, Energies, structures, and electronic properties of molecules in solution with the C-PCM solvation model. *J. Comput. Chem.* **24**, 669–681 (2003).

38. G. Scalmani, M. J. Frisch, Continuous surface charge polarizable continuum models of solvation. I. General formalism. *J. Chem. Phys.* **132**, 114110 (2010).

39. F. Lipparini, G. Scalmani, B. Mennucci, E. Cancès, M. Caricato, M. J. Frisch, A variational formulation of the polarizable continuum model. *J. Chem. Phys.* **133**, 014106 (2010).

40. F. Eckert, A. Klamt, Fast solvent screening via quantum chemistry: COSMO-RS approach. *AIChE J.* **48**, 369–385 (2002).

41. A. Klamt, The COSMO and COSMO-RS solvation models. *Wiley Interdiscip. Rev. Comput. Mol. Sci.* **1**, 699–709 (2011).

42. A. Klamt, M. Diedenhofen, Calculation of solvation free energies with DCOSMO-RS. *J. Phys. Chem. A* **119**, 5439–5445 (2015).

43. A. Hellweg, F. Eckert, Brick by brick computation of the gibbs free energy of reaction in solution using quantum chemistry and COSMO-RS. *AIChE J.* **63**, 3944–3954 (2017).

44. R. Ahlrichs, M. Bär, M. Häser, H. Horn, C. Kölmel, Electronic structure calculations on workstation computers: The program system turbomole. *Chem. Phys. Lett.* **162**, 165–169 (1989).

45. O. Treutler, R. Ahlrichs, Efficient molecular numerical integration schemes. *J. Chem. Phys.* **102**, 346–354 (1995).

46. M. V. Arnim, R. Ahlrichs, Performance of parallel TURBOMOLE for density functional calculations. *J. Comput. Chem.* **19**, 1746–1757 (1998).

47. A. D. Becke, Density-functional exchange-energy approximation with correct asymptotic behavior. *Phys. Rev. A* **38**, 3098–3100 (1988).

48. J. P. Perdew, Density-functional approximation for the correlation energy of the inhomogeneous electron gas. *Phys. Rev. B.* **33**, 8822–8824 (1986).

49. F. Weigend, R. Ahlrichs, Balanced basis sets of split valence, triple zeta valence and quadruple zeta valence quality for H to Rn: Design and assessment of accuracy, *Phys. Chem. Chem. Phys.* **7**, 3297–3305 (2005).

50. K. Eichkorn, O. Treutler, H. Öhm, M. Häser, R. Ahlrichs, Auxiliary basis sets to approximate Coulomb potentials. *Chem. Phys. Lett.* **242**, 652–660 (1995).

51. K. Eichkorn, F. Weigend, O. Treutler, R. Ahlrichs, Auxiliary basis sets for main row atoms and transition metals and their use to approximate Coulomb potentials. *Theor. Chem. Acc.* **97**, 119–124 (1997).

52. F. Weigend, Accurate Coulomb-fitting basis sets for H to Rn. *Phys. Chem. Chem. Phys.* **8**, 1057–1065 (2006).

53. M. Sierka, A. Hogekamp, R. Ahlrichs, Fast evaluation of the Coulomb potential for electron densities using multipole accelerated resolution of identity approximation. *J. Chem. Phys.* **118**, 9136–9148 (2003).

54. A. Klamt, M. Diedenhofen, A refined cavity construction algorithm for the conductor-like screening model. *J. Comput. Chem.* **39**, 1648–1655 (2018).

55. S. Grimme, J. Antony, S. Ehrlich, H. Krieg, A consistent and accurate ab initio parametrization of density functional dispersion correction (DFT-D) for the 94 elements H-Pu. *J. Chem. Phys.* **132**, 154104 (2010).

56. NIST Chemistry WebBook, Sadtler Research Labs Under US-EPA Contract (10 February 2021); https://webbook.nist.gov/cgi/cbook.cgi?ID=C103719&Type=IR-SPEC&Index=0#IR-SPEC.
